# Binding, Sensing, And Transporting Anions with Pnictogen Bonds: The Case of Organoantimony Lewis Acids

**DOI:** 10.1021/jacs.3c06991

**Published:** 2023-08-30

**Authors:** Brendan L. Murphy, François P. Gabbaï

**Affiliations:** Department of Chemistry, Texas A&M University, College Station, Texas 77843-3255, United States

## Abstract

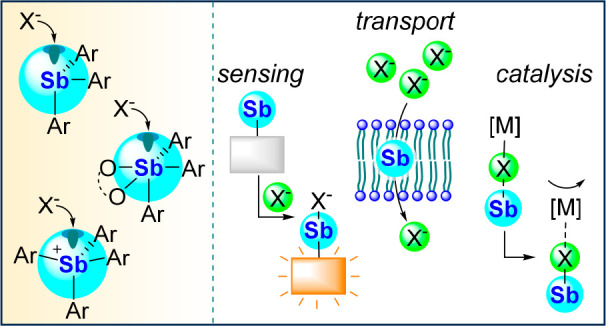

Motivated by the discovery of main group Lewis acids that could compete or possibly outperform the ubiquitous organoboranes, several groups, including ours, have engaged in the chemistry of Lewis acidic organoantimony compounds as new platforms for anion capture, sensing, and transport. Principal to this approach are the intrinsically elevated Lewis acidic properties of antimony, which greatly favor the addition of halide anions to this group 15 element. The introduction of organic substituents to the antimony center and its oxidation from the + III to the + V state provide for tunable Lewis acidity and a breadth of applications in supramolecular chemistry and catalysis. The performances of these antimony-based Lewis acids in the domain of anion sensing in aqueous media illustrate the favorable attributes of antimony as a central element. At the same time, recent advances in anion binding catalysis and anion transport across phospholipid membranes speak to the numerous opportunities that lie ahead in the chemistry of these unique main group compounds.

## Introduction

Lewis acidic main group compounds have become ubiquitous in both organic and organometallic chemistry as illustrated by the rise perfluorinated triarylboranes^1^ and their prevalence in numerous applications.^2^ Even if much less reactive than their boron halide counterparts, these organoboranes are mostly limited to use in organic media and often readily decompose in aqueous environments. These water compatibility issues can be resolved, at least in part, by using aryl groups like mesityls to sterically protect the boron center against water.^3^ Using this strategy, several groups, including ours, have prepared water-compatible boranes^4^ whose solubilities can be enhanced by the introduction of peripheral cationic moieties.^5^ These cationic groups can also increase the anion affinity of these boranes, a feature that we have exploited for the sensing of toxic anions such as fluoride and cyanide in aqueous solutions.^6^ While these boranes can be used to detect parts-per-million (ppm) concentrations of these anions, the binding event is typically accompanied by a fluorescence “turn-off” response^7^ thereby restricting their analytical practicality as molecular sensors. These boranes also tend to decompose upon standing in aqueous media over time, and their vulnerability to oxidation^8^ challenges their longevity under aerobic conditions, especially in the presence of reactive oxygen species.

**Scheme 1:**
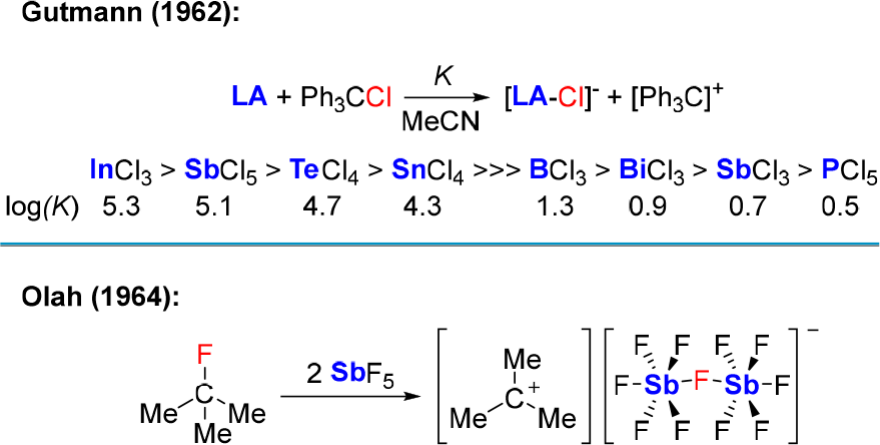
Classic Investigations into the Lewis Acidity of Antimony Compounds

However, as documented in the early work of Gutmann, Lewis acidity also emerges as a prevalent property of Sb(V) halides. Using the activation of the C–Cl bond of trityl chloride in MeCN as an indicator, Gutmann demonstrated that SbCl_5_ stands near the apex of his chloride ion affinity scale, second only to InCl_3_ (Scheme 1).^9^ In another series of seminal contributions, Olah,^10^ and later Gillespie,^11^ showed that SbF_5_ could be used for the activation of C–F bonds in organic compounds (Scheme 1) or for the generation of “superacids”.^12^ As seen in boron chemistry, early reports also suggested that organoantimony derivatives retain some of the Lewis acidic characteristics of their halide analogs while displaying much greater tolerance to ambient air and water.^13^ Encouraged by these precedents, we posited that such compounds may be uniquely suited for anion binding in a variety of competitive media.

Building off these seminal studies, we have undertaken extensive investigations into the properties and applications of organoantimony compounds in our laboratory. In this Perspective, we will review these efforts while spotlighting other notable works also focused on the development of Lewis acidic organoantimony compounds as new anion binding and trafficking platforms. In doing so, we will first discuss the structural and electronic factors that grant antimony its Lewis acidity and show how these basic principles have guided the design of organoantimony-based anion binding units and sensors. We will also highlight the arising utilities of organoantimony compounds for anion-binding catalysis and as transporters of anions across biological membranes. Finally, because this Perspective will only discuss organic derivatives of antimony, we direct the reader’s attention to our adjacent work employing antimony as a coordination non-innocent ligand for late transition metal centers, in which the antimony center is also capable of anion binding^14^ and sensing.^15^

## Origin of Lewis Acidity in Simple Antimony Compounds and Related Concepts

### The Case of Antimony(III) Derivatives

**Figure 1:**
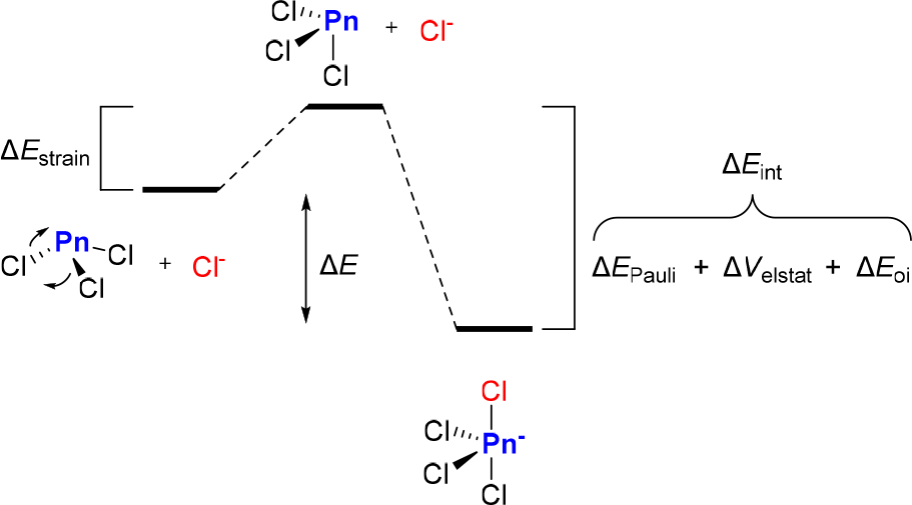
Illustration of the structural and energetic components of chloride binding by Pn(III) centers investigated by Bickelhaupt and co-workers.

To understand the origin of the elevated Lewis acidity of antimony compounds compared to lighter group 15 elements, or pnictogens (Pn), we look back to the experimental work of Gutmann,^16^ who interrogated the conversion of pnictogen trichlorides (PnCl_3_) into the corresponding tetrachloropnictate complexes.^9^ These pioneering studies showed that the chloride affinity of SbCl_3_ exceeds that of AsCl_3_ and far exceeds that of PCl_3_ in MeCN.^9^ These experimental results are corroborated by the computational work of Bickelhaupt and co-workers who applied the activation strain model to clarify the origin of this simple trend.^17^ This computational approach, as summarized in Figure 1,^18^ considers the formation of a complex based on: (i) the deformation of the interacting partners to geometries that match those in the final complexes; (ii) the formation of the final complex by association of the deformed partners. These two steps give rise to two energy terms, referred to as the strain energy (Δ*E*_strain_) and the interaction energy (Δ*E*_int_), respectively. Furthermore, energy decomposition analysis (EDA) schemes can be used to shed light on how electrostatic forces (Δ*V*_elstat_), orbital-based interactions (Δ*E*_oi_), and Pauli repulsions (Δ*E*_Pauli_) influence Δ*E*_int_ (Table 1).

**Table 1:** Energy Decomposition Analysis of Representative Cl^–^ → PnCl_3_ Interactions^17^^a^

PnB Interaction	Δ*E*_strain_	Δ*E*_Pauli_	Δ*V*_elstat_	Δ*E*_oi_	Δ*E*_int_	Δ*E*
Cl^–^→PCl_3_	14.4	119.2	–84.8	–74.2	–39.8	–25.5
Cl^–^→AsCl_3_	11.3	101.9	–85.5	–62.8	–46.4	–35.1
Cl^–^→SbCl_3_	8.9	100.9	–93.3	–59.2	–51.7	–42.7

a Values are given in kcal·mol^–1^.

First, the Δ*E*_strain_ needed to distort the Pn(III) center from its ground state geometry to that adopted in the tetrachloropnictate anion is the lowest for the antimony derivative. This lower value can be explained by the Cl–Sb–Cl angles in SbCl_3_ which are close to 90°, as in the corresponding antimonate complex. A slight decrease is also seen in Δ*E*_Pauli_, reflecting the larger size of antimony and its ability to more easily accommodate an extra ligand. A more important factor is the electrostatic term (Δ*V*_elstat_). The magnitude of this term notably increases down the group as the electropositivity and polarizability of the Pn center also increase. It follows that [SbCl_4_]^−^ is the most electrostatically stabilized complex of the series. The Δ*E*_oi_ term is not to be ignored though. Because of the increasing diffuseness and radial nodes of its valence orbitals, the covalency of the newly formed Pn-Cl bond decreases going down the group. Inspection of the values in Table 1 shows that this decrease is moderate with the Δ*E*_oi_ of the antimony system being only marginally more positive than that for arsenic. Altogether, out of the series considered, SbCl_3_ binds to chloride most exothermically. When compared to the arsenic system, the superior Lewis acidity of SbCl_3_ can be seen as resulting mostly from the more negative Δ*V*_elstat_ term, with all other terms offsetting one another (Table 1).

### The Case of Antimony(V) Derivatives

The early work of Gutmann summarized in Scheme 1 showed that antimony(V) derivatives are more Lewis acidic than their trivalent counterparts.^19^ The same study found that SbCl_5_ is more chloridophilic than PCl_5_ by nearly 5 orders of magnitude,^9^ again suggesting that Lewis acidity increases as the group is descended. This conclusion is supported by several computational studies which show that the computed gas-phase fluoride ion affinities (FIAs) of the pnictogen pentafluorides increase from PF_5_ to SbF_5_ (Table 2).^20^

**Table 2:** Computed Gas-Phase FIAs of Pnictogen Pentafluorides^a^

Pnictogen pentafluoride	FIA (Greb and co-workers)^20a^	FIA (Rabe and Krossing)^20b^	FIA (Moc and Morokuma)^20c^
PF_5_	384	394	385
AsF_5_	439	426	436
SbF_5_	496	489	492

a Values are given in kJ·mol^–1^.

A recent activation-strain and EDA study investigated fluoride anion binding by Pn(V) derivatives.^21^ The data in Table 3, compiled for the PnF_5_ species, show that the Δ*E*_oi_ and Δ*V*_elstat_ terms become less negative as the group descends because of the lengthening of the Pn-F linkage and the increased diffuseness of the Pn orbitals. The decrease of these stabilizing interactions is compensated for by the Pauli repulsions which become lower as the size of Pn increases. Owing to the balancing of these opposite influences, Δ*E*_int_ shows little variation down the group. Thus, through the lens of the activation-strain model, the high Lewis acidity of antimony originates from the lower Δ*E*_strain_ term, which reflects the greater flexibility of SbF_5_. With longer and more diffuse Pn-F σ bonds, the fluoride ligands acquire greater freedom of motion and stand further from one another, allowing the SbF_5_ unit to more easily accommodate the *C*_4*v*_ geometry it displays in [SbF_6_]^−^.

**Table 3:** Energy Decomposition Analysis of Representative F^–^ → PnF_5_ Interactions^21^^a^

PnB Interaction	Δ*E*_strain_	Δ*E*_Pauli_	Δ*V*_elstat_	Δ*E*_oi_	Δ*E*_int_	Δ*E*
F^–^→PF_5_	51.9	230.9	–220.1	–154.1	–143.4	–91.6
F^–^→AsF_5_	33.0	207.5	–215.1	–129.8	–137.5	–104.5
F^–^→SbF_5_	23.7	166.7	–209.3	–101.4	–144.1	–120.3

a Values are given in kcal·mol^–1^.

### Is a group 15 Lewis acid the same thing as a pnictogen bond donor?

**Figure 2:**
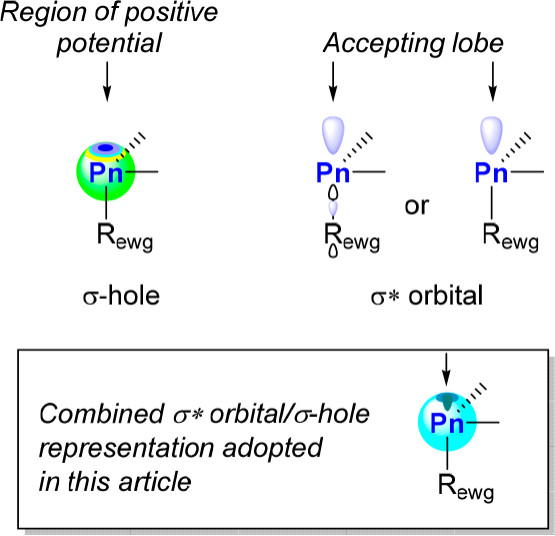
Schematic representations of the “σ hole” (left) and the σ* orbital of a trivalent pnictogen. The σ* orbital is presented in a simplified fashion, with the second representation only keeping the accepting lobe. A similar picture could be drawn for a pentavalent pnictogen. R_ewg_ is an electron-withdrawing group.

We note that donor–acceptor interactions involving Pn derivatives as acceptors have been known for several decades and only recently referred to as “pnictogen bonds” (PnBs).^22^ Akin to the halogen bond^23^ and the chalcogen bond,^24^ PnBs are often described as resulting from the interaction of a donor (D) with a Pn-centered electrophilic “σ hole”, a region of positive electrostatic potential opposite to one of the primary bonds (Figure 2).^22,25^ In this light, the PnB is often regarded as a “non-covalent” interaction. While such a description may be fitting of cases where the interaction is weak, it presents the danger of oversimplifying the nature of PnBs. Namely, this characterization ignores the contribution of orbital-based interactions, like those identified in the above-mentioned work of Bickelhaupt.^18^ Such orbital-based interactions were discussed in no uncertain terms in the early work of Alcock^26^ who in 1972 identified the ability of main group element-centered σ* orbitals to accept electron density from filled donor orbitals (Figure 2). This concept was revisited by Norman in the specific case of pnictogen derivatives in 1994.^27^ This notion is also prevalent in the seminal work of Scheiner from 2011 on the weakly associated H_3_N → PH_3_ adduct in which the nitrogen lone pair aligns with one of the primary P–H bonds and transfers some of its electron density into the corresponding P–H σ* lobe.^28^ The importance of such orbital or charge transfer interactions will depend on the nature of both the donor and the Pn acceptor. It can be weak as in the H_3_N → PH_3_ adduct or strong as in X^–^ → PnX_3_ adducts, in which the newly formed X^–^ → Pn bond displays considerable covalency. It follows that the “non-covalent” adjective as a descriptor of PnBs loses its legitimacy.^18^ PnBs are simply examples of dative bonds,^29^ connecting a donor to a group 15 acceptor ([Pn]), stabilized through the interplay of Coulombic and covalent or orbital-based interactions with possible contribution from dispersion interactions. We therefore consider that any D → [Pn] complex can also be described as a Lewis adduct and a PnB donor as a Lewis acid.

## Anion Complexation Chemistry

### Antimony(III) Compounds

**Scheme 2:**
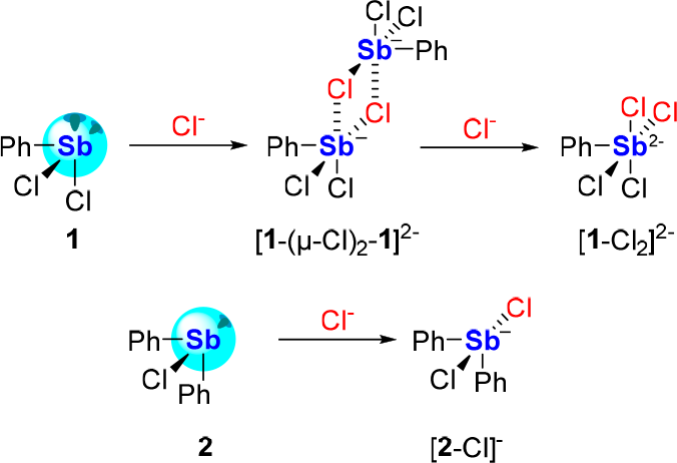
Chloride Complexation by Chlorostibines **1** and **2**

The Lewis acidity of antimony persists despite the passivating effect of an aryl appendage. Indeed, simple arylantimony(III) dihalide species are sufficiently Lewis acidic to form isolable antimonate complexes. Such is the case of PhSbCl_2_ (**1**), which exists in the solid-state as a weak contact aggregate^30^ and reacts with chloride to form the bridged dianion ([**1**-(μ-Cl)_2_-**1**]^2–^, Scheme 2).^31^ Each antimony(III) center of this centrosymmetric dimer adopts a local square pyramidal geometry, with pairs of chloride ligands in *trans* positions to each other. The disposition of these two chloride ligands can be viewed as resulting from their addition to the sites featuring the deepest σ holes and the most energetically accessible σ* orbitals. Moreover, treating **1** with 2 equiv of chloride gives rise to the monomeric, square pyramidal-shaped dianion [**1**-Cl_2_]^2–^, further indicating that both binding sites are accessible to an incoming chloride.^31a^

Similarly, diarylantimony(III) halide species have also been shown to display notable Lewis acidity, and are also capable of Lewis base recognition at a binding site that is also *trans* to the halide ligand.^32^ For example, the monomeric Ph_2_SbCl (**2**)^33^ also reacts with chloride anions to form [Ph_2_SbCl_2_]^−^ ([**2**-Cl]^−^, Scheme 2), which takes on a seesaw geometry with the two chloride ligands *trans* to each other.^31a,34^ To the best of our knowledge, the dianionic [**2**-Cl_2_]^2–^ has never been isolated, pointing to the inaccessibility of the σ hole/σ*-orbital *trans* to a phenyl group.

**Figure 3:**
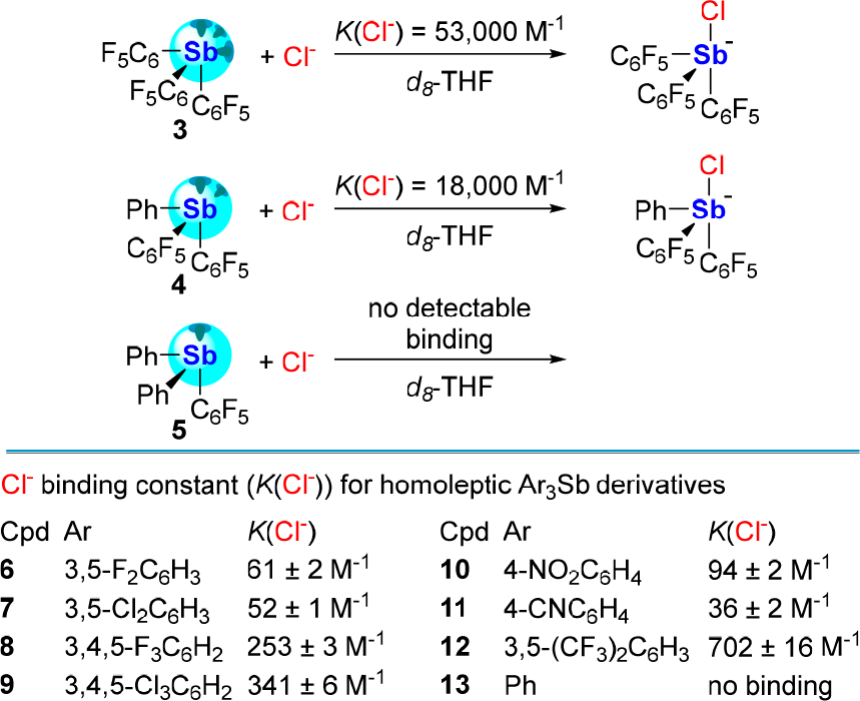
Top: Chloride binding constants of stibines **3**–**5** as determined by Matile.^36^ Bottom: Chloride binding constants of homoleptic stibines **6**–**13** as determined by Beer.

Complete arylation of the antimony(III) center typically limits the chemistry of triarylstibines to use as L type ligands in coordination chemistry.^35^ However, the introduction of electron-withdrawing aryl groups can elicit PnB donor properties at the antimony center. Such is the case of (C_6_F_5_)_3_Sb (**3**), which forms a structurally characterized adduct with Ph_3_PO.^19^ The Lewis acidic behavior of **3** also manifests in its affinity for chloride anions, as reported by Matile.^36^^19^F NMR titration with tetrabutylammonium chloride (TBACl) in *d*_8_-THF affords a chloride association constant (*K*(Cl^–^)) of 53,000 M^–1^. Lower chloride binding is observed in the case of Ph(C_6_F_5_)_2_Sb (**4**, *K*(Cl^–^) = 1,800 M^–1^), while Ph_2_(C_6_F_5_)Sb (**5**) shows no measurable affinity for the ionic guest, pointing to the crucial role played by the number of pentafluorophenyl substituents appended to the antimony center (Figure 3).

Recent work by the group of Beer further illustrates the benefits resulting from introducing electron-withdrawing units on Ar_3_Sb systems (Figure 3).^37^ Indeed, **6**–**12** bind chloride in *d*_8_-THF as indicated by ^1^H NMR spectroscopy. Chloride binding peaks in the case of **12**, which bears the most electron deficient aryl rings of the compounds studied. Moreover, the chloride affinity of **12** was markedly higher than its affinity for Br^–^, I^–^, and NO_3_^–^, and more basic anions such as OAc^–^, OCN^–^, and NO_2_^–^ were found to bind more weakly than Cl^–^. We will note in passing a possible disconnect in the magnitude of the chloride binding constants measured by the Matile and Beer groups since the C_6_F_5_ and the 3,5-(CF_3_)_2_C_6_H_3_ aryl groups have similar electron-withdrawing properties when appended to main group Lewis acids.^19^ It is also worth noting that Ph_3_Sb (**13**) showed no measurable affinity for chloride in *d*_8_-THF.

**Figure 4:**
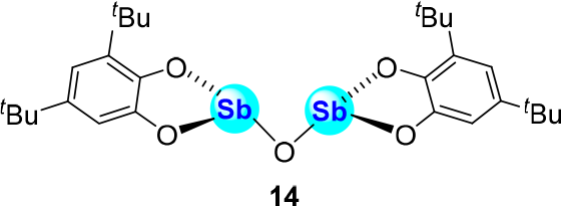
Structure of bidentate distibine **14**.

Anion binding has also been investigated by the Cozzolino group using oxygen-bridged bidentate distibines^38^ such as **14** which displays halide binding constants in CH_2_Cl_2_ of 870,000 ± 98,000 M^-1^, 16,900 ± 5,000 M^–1^, 2,390 ± 1,470 M^–1^ for chloride, bromide, and iodide, respectively (Figure 4).^39^ In addition to showing halide anion binding selectivity, **14** also interacts with the cyanide anion and effectively promotes its phase transfer for applications in cyanation chemistry.

### Antimony(V) Compounds – Halostiboranes

**Figure 5:**
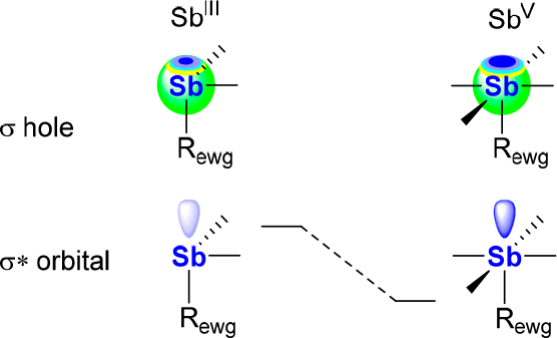
Deepening of the σ hole and stabilization of the σ* orbital resulting from oxidation of an Sb(III) species to an Sb(V) species.

Harkening back to initial studies on the anion binding chemistry of antimony (Scheme 1), Gutmann found that SbCl_5_ possesses a chloridophilicity that is nearly 5 orders of magnitude greater than that of SbCl_3_.^9^ The increased Lewis acidity observed upon oxidation can be correlated to a deepening of the σ hole at antimony as well as a lowering in the energy of the σ* orbital aligned with an incoming Lewis base, as illustrated in Figure 5.^19^ These effects, which have been examined in the special case of **3** and its corresponding tetrachlorocatecholatostiborane (*vide infra*),^19^ should apply to any pair of antimony Lewis acids differentiated by the +III or +V oxidation state of the antimony center.

**Scheme 3:**
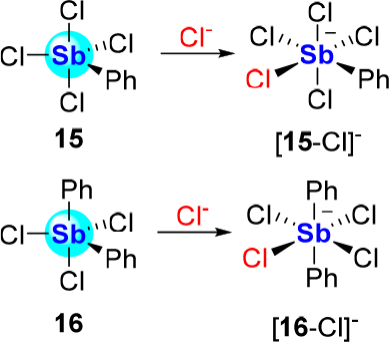
Chloride Complexation by Halostiboranes **15** and **16**

The Lewis acidity of the Sb(V) compounds can be observed in arylated derivatives such as PhSbCl_4_ (**15**) and Ph_2_SbCl_3_ (**16**), which readily form the corresponding chloroantimonate species [PhSbCl_5_]^−^ ([**15**-Cl]^−^)^40^ and [Ph_2_SbCl_4_]^−^ ([**16**-Cl]^−^)^41^ when treated with an appropriate chloride salt (Scheme 3). The formation of these salts may be seen as mirroring that of their +III analogues, namely, the [PhSbCl_3_]^−^ anion ([**1**-Cl]^−^) and [Ph_2_SbCl_2_]^−^ anion ([**2**-Cl]^−^) (Scheme 2). Even if direct comparisons of these Lewis acids are not available in the literature, antimony(V) derivatives **15** and **16** should display greater Lewis acidic properties than their trivalent counterparts.

**Scheme 4:**
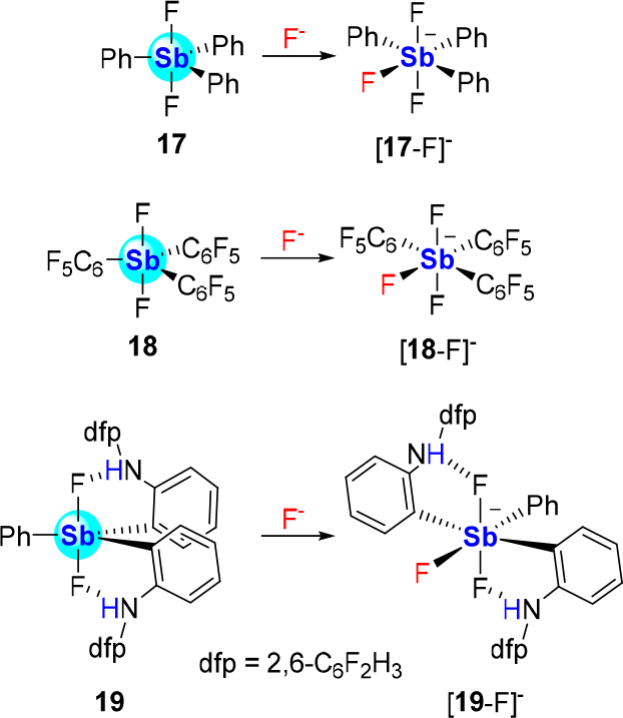
Fluoride Complexation by Triaryldihalostiboranes **17**–**19**

Further substitution of the antimony center by aryl substituents can be anticipated to negatively affect its Lewis acidity. These deleterious effects are already evident when analyzing the behavior of Ph_3_SbCl_2_, which to our knowledge, does not associate with chloride ions. On the other hand, Ph_3_SbF_2_ (**17**), which contains smaller and more electron-withdrawing ligands, complexes fluoride in CDCl_3_ to form [Ph_3_SbF_3_]^−^ ([**17**-F]^−^, Scheme 4) as verified by ^19^F NMR.^42^ A stronger complexation was observed in the case of (C_6_F_5_)_3_SbF_2_ (**18**), leading to the unambiguous characterization of [(C_6_F_5_)_3_SbF_3_]^−^ ([**18**-F]^−^) formed by addition of CsF to **18** in MeOH.^43^ Another strategy to elevate the anion affinity of triaryldihalostiboranes rests on the introduction of hydrogen bonding (HB) groups, as in the case of (**19**), a complex featuring two 2,6-difluoro-*N*-phenylbenzenamine groups. Our investigation of this derivative shows that it outcompetes Ph_3_SbF_2_ for fluoride binding in CDCl_3_ at −30 °C.^42^

**Scheme 5:**
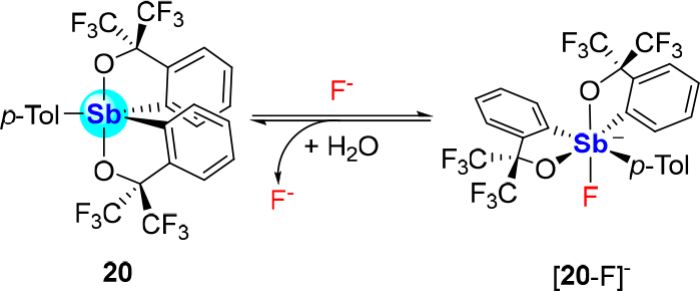
Fluoride Binding by Stiborane **20**

Anion complexation could also be achieved by pentacoordinate antimony(V) systems in which the antimony center is bound to two doubly deprotonated 2,2,2,2′,2′,2′-hexafluorocumylalcohol C,O-bidendate ligands.^44^ Indeed, antimony(V) derivative **20** was shown to complex fluoride anions, as verified by the structural characterization of [**20**-F][Et_4_N]. Interestingly, variable-temperature ^19^F NMR studies reveal that [**20**-F]^−^ exists as several structural isomers at −40 °C, suggesting that the fluoride anion can reversibly sample the various anion binding sites presented by **20** (Scheme 5). Addition of water to an acetone solution of [**20**-F]^−^ induces decomplexation of the fluoride anion, reminding us of its high hydration energy (−504 kJ·mol^–1^).

### Antimony(V) Compounds - Catecholatostiboranes

#### Monofunctional Systems

**Scheme 6:**
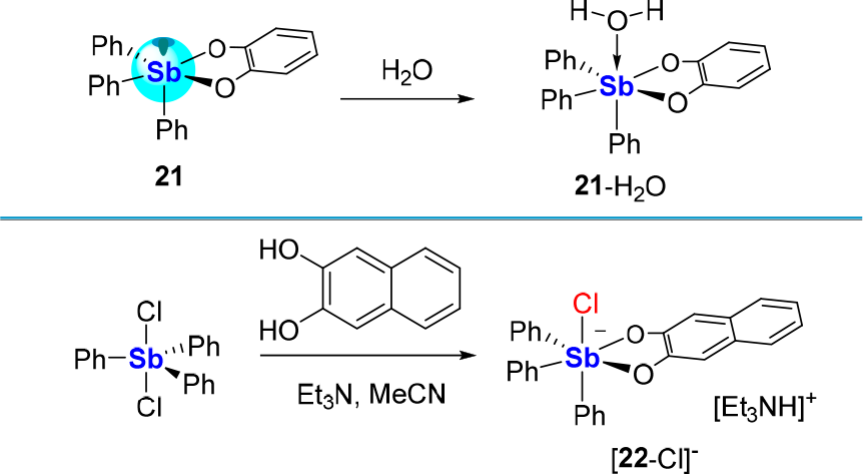
Lewis Acidic Behaviors of Catecholatostiborane **21** and **22**

The imposition of geometrical constraints is also an effective way to enhance the Lewis acidity of antimony derivatives. One of the earliest pieces of evidence for these effects was provided in the case of Ph_3_SbO_2_C_6_H_4_**(21**, Scheme 6) that, based on X-ray analysis, engages a water molecule *via* formation of an O → Sb bond of 2.51 Å.^13b^ The formation of such a water adduct is not observed for Ph_3_SbF_2_ and Ph_3_SbCl_2_. This contrasting behavior points to the role played by structural constraints imposed by the SbO_2_C_2_ five-membered ring which elevates the Lewis acidity of the antimony(V) center *via* distortion of its ground state geometry. Another favorable feature of catecholate groups is their minimal steric profile since the coordinating oxygen ligands extend out from the aromatic backbone and thus partly escape its steric hindrance. The formation of [**22**-Cl][Et_3_NH] by reaction of Ph_3_SbCl_2_ with 2,3-naphthalenediol and Et_3_N is another important precedent directly relevant to the topic of this Perspective since it involves the complexation of a halide anion (Scheme 6).^45^ The formation of [**22**-Cl]^−^ contrasts with the absence of reactivity of Ph_3_SbCl_2_ toward the chloride anion.

**Figure 6:**
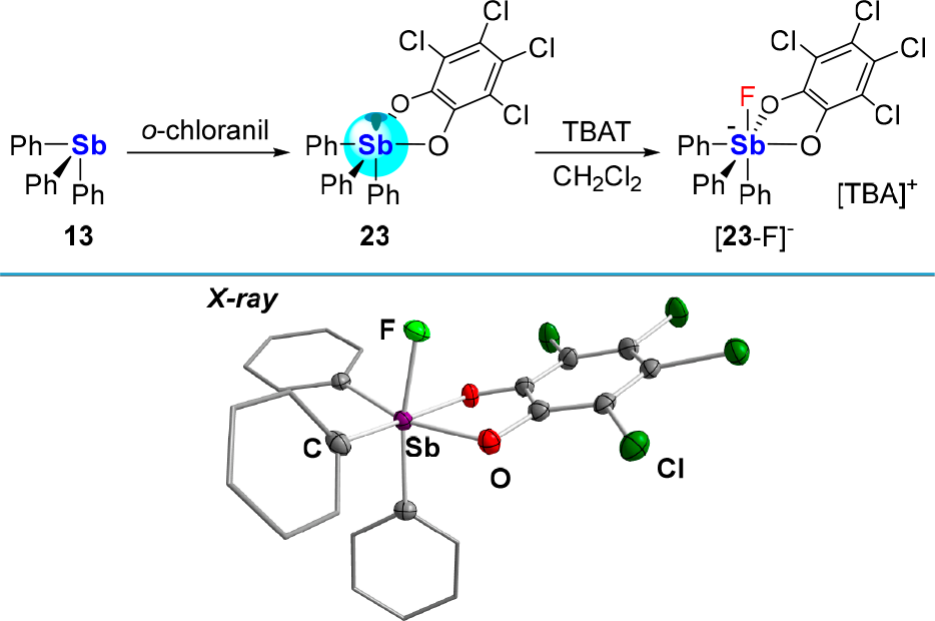
Top: Oxidation of stibine **13** following treatment with *o*-chloranil to yield catecholatostiborane **23**, and isolation of [**23**-F][TBA] upon treatment of **23** with TBAT. Bottom: Solid-state structure of [**23**-F]^−^.

In addition to their elevated Lewis acidity, catecholatostiboranes also benefit from their ease of preparation. In addition to metathesis reactions such as that used to form **22**, these species can also be obtained through the simple combination of a triarylstibine with an *ortho*-quinone such as *o*-chloranil. Owing to its perchlorination, this quinone is a particularly potent oxidant that engages a range of organoantimony compounds, including **13** which is readily converted into Ph_3_SbO_2_C_6_Cl_4_ (**23**, Figure 6) as demonstrated by Holmes in 1987.^45^ Our reinvestigation of this compound shows that unlike its trivalent precursor, it behaves as a potent Lewis acid, able to bind a fluoride anion when treated with [TBA][Ph_3_SiF_2_] (TBAT). The formation of [**23**-F][TBA] took place in CH_2_Cl_2_ and survived aqueous workup, speaking to the stability of the Sb–F bond.^46^ The solid-state structure of [**23**-F]^−^ displays a short Sb–F bond distance of 1.9877(13) Å (Figure 6), only slightly longer than the average Sb–F bond length in [SbF_6_]^−^ (1.844 Å).^47^ As noted at the start of this section, we have explained the increased Lewis acidity observed upon oxidation by a lowering of the σ* orbital and a deepening of the σ hole.^19^ These effects, combined with the structural predisposition resulting from the presence of a SbO_2_C_2_ five-membered ring, may serve to rationalize the anion affinity of molecules such as **23**.

**Figure 7:**
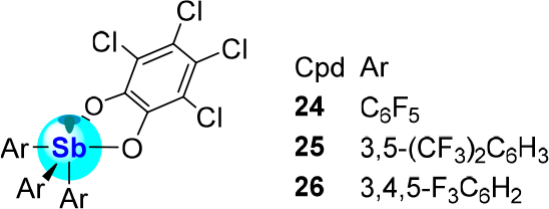
Structures of catecholatostiboranes **24**–**26**.

Altogether, the conversion of **13** into **23** establishes a simple approach for converting potentially Lewis basic triarylstibines to Lewis acidic stiboranes. We found that even electron poor stibines such as (C_6_F_5_)_3_Sb (**3**) and (3,5-(CF_3_)_2_C_6_H_3_)_3_Sb (**12**) could be converted into the corresponding tetrachlorocatecholatostiboranes **24** and **25** (Figure 7).^19^ As is the case for the parent triphenyl derivative, the antimony-centered LUMO is stabilized by 0.89 and 0.78 eV upon formation of **24** and **25**, respectively. A sizable increase in the FIA values (δ(FIA)) was also computed for these three pairs of compounds upon oxidation of the antimony center. The largest change was observed in the case of the parent derivative **13** whose FIA increases by δ(FIA) = 150 kJ·mol^–1^ upon conversion into **23** versus 111 kJ·mol^–1^ and 132 kJ·mol^–1^ for the **3**/**24** and **12**/**25** couples, respectively. This strategy has been applied by Matile on other fluorinated triarylstiboranes including (3,4,5-F_3_C_6_H_2_)_3_Sb (**8**), which was readily converted into the corresponding tetrachlorocatecholatostiborane **26**.^48^ We should also acknowledge the growing role that perhalogenated catecholate groups are playing in the chemistry of arsenic,^49^ silicon,^50^ germanium,^51^ and phosphorus Lewis acids.^49,52^

**Figure 8:**
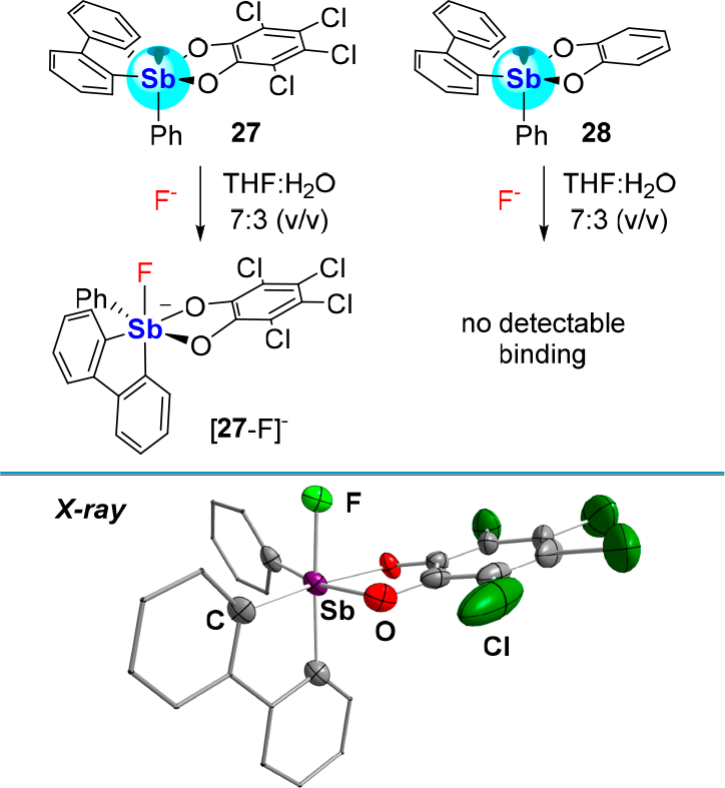
Top: Fluoride binding by catecholatostiboranes **27** and **28** in THF:water (7:3 (v/v)). Bottom: Solid-state structure of [**27**-F]^−^.

We also aimed to assess the influence of catecholate perchlorination on the Lewis acidity of the antimony center. For example, we decided to compare the anion affinity of **27**(45) to that of the perprotiocatecholate analogue **28**. We found that **27** displays a high binding constant for the fluoride anion (*K*(F^–^) of 13,500 ± 1,400 M^–1^ in THF:water (7:3 (v/v)) as established by a UV–vis titration while **28** shows no affinity for the anion under the same conditions (Figure 8).^53^ The contrasting behaviors of these two compounds underscore how important the electronic features of the catecholate are in governing the anion affinity of these compounds. In this case, perchlorination of the catecholate most likely reduces the donicity of the oxygen atoms, leaving a more exposed and thus more Lewis acidic antimony(V) center. The anionic complex [**27**-F]^−^, which has been characterized as a tris(dimethylamino)sulfonium ([TAS]^+^) salt, displays a short Sb–F bond length of 1.973(4) Å, on par with that of the aforementioned [**23**-F]^−^.^53^ In addition to demonstrating the importance of catecholate perchlorination, compound **27** may also benefit from its bicyclic structure. The integration of two antimony-fused five membered rings most likely accentuates the geometrical constraints imposed on the ground state structure, boosting its Lewis acidity.^54^

## Bifunctional Systems

**Figure 9:**
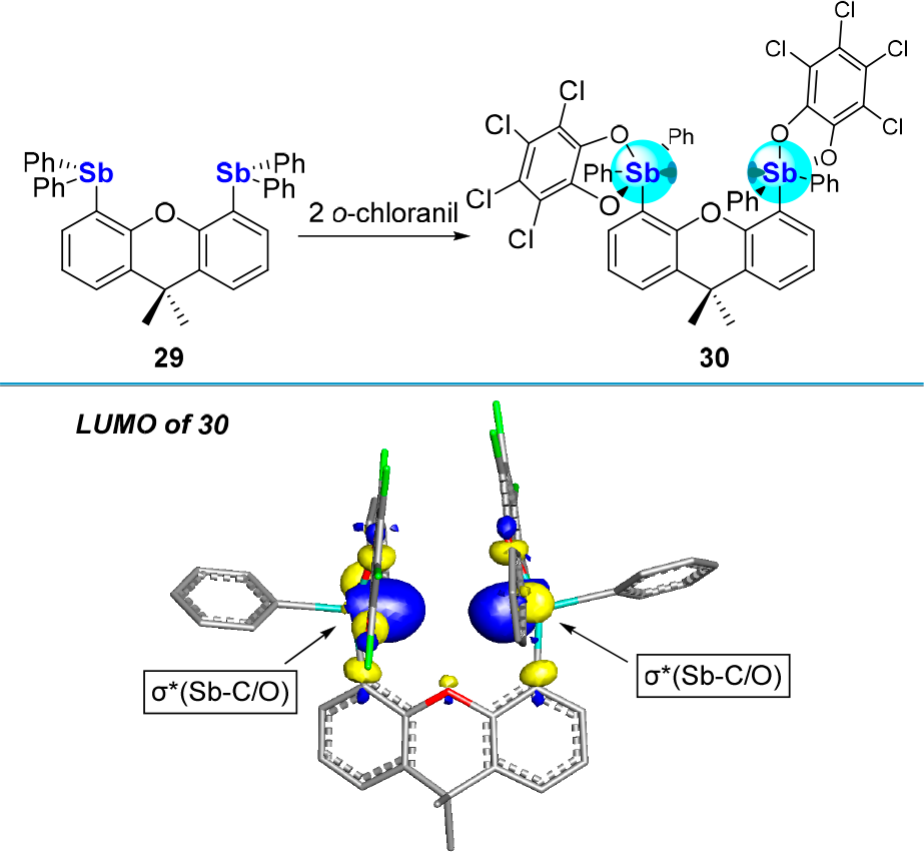
Top: Oxidation of distibine **29** to distiborane **30** following treatment with two equiv of *o*-chloranil. Bottom: Contour plot of the LUMO (isovalue: 0.05) of **30** showing the projection of the σ*(Sb–C/O) orbitals into the binding pocket.

The facility by which tetrachlorocatecholatostiboranes can be prepared prompted us to investigate their introduction in more elaborated scaffolds, including bifunctional ones that would support anion chelation.^4b,4c,6,55^ To explore this possibility, we synthesized the 4,5-bis(diphenylantimony)-9,9-dimethylxanthene (**29**) and found that it could be easily converted into **30** (Figure 9).^46^ A structural analysis of **30** shows that the two square-pyramidal stiborane units orient their basal square face inward, thus defining a binding pocket possibly adapted to the capture of small anions. Another favorable feature of this derivative lies in the nature of its LUMO, which spans the two antimony atoms and displays significant σ*(Sb–C/O) parentage with lobes extending toward the center of the binding pocket.

**Figure 10:**
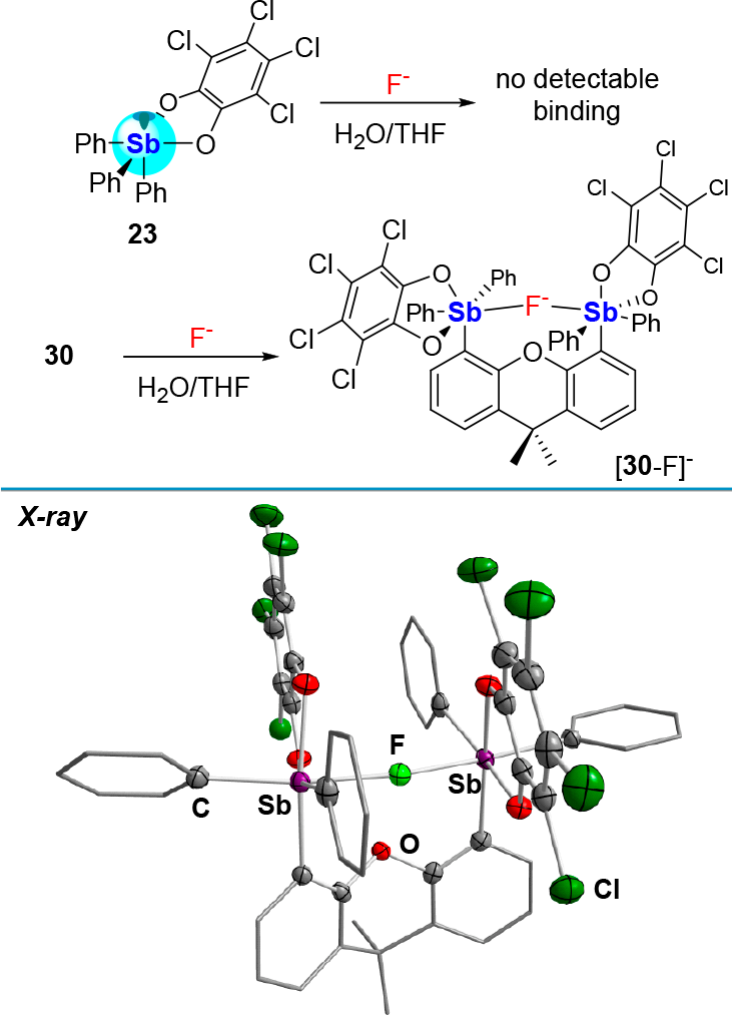
Top: Fluoride binding by **23** and **30** in water:THF (9.5:0.5 (v/v), 10 mM citrate, and 45 mM Triton X-100). Bottom: Solid-state structure of [**30**-F]^−^.

Congruent with these attributes, **30** readily complexes a fluoride anion to afford [**30**-F]^−^ (Figure 10). A crystal structure of its [TBA]^+^ salt confirms the formation of a fluoride chelate complex supported by two Sb–F bonds of 2.1684(19) Å and 2.1621(19) Å.^46^ This distance shows a measurable elongation compared to the value of 1.9877(13) Å found for the terminal Sb–F bond in [**23**-F]^−^, in line with the μ_2_ coordination mode of the fluoride anion in [**30**-F]^−^. The thermodynamic impact of anion chelation manifests in the acidity constant (p*K*_Sb_) of the antimony center, which is equivalent to the pH at which 50% of the antimony Lewis acid is neutralized by a hydroxide anion. Indeed, the p*K*_Sb_ of **30** (5.8 ± 0.1) measured in water:THF (9.5:0.5 (v/v), 10 mM sodium phosphate, 45 mM Triton X-100) is significantly lower than that of **23** (7.4 ± 0.1). In line with this result, whereas **30** is capable of binding fluoride in a highly competitive water:THF (9.5:0.5 (v/v), pH 4.34, 10 mM citrate, 45 mM Triton X-100) solution with a *K*(F^–^) value of 700 ± 30 M^–1^, **23** does not engage the fluoride anion in that medium.

**Scheme 7:**
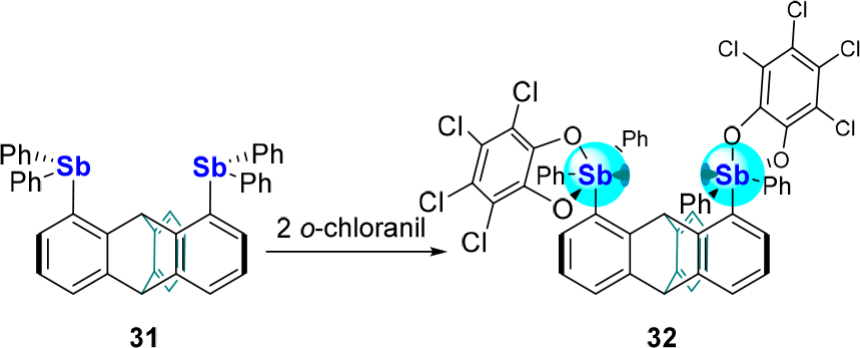
Oxidation of Distibine **31** into Distiborane **32**

**Figure 11:**
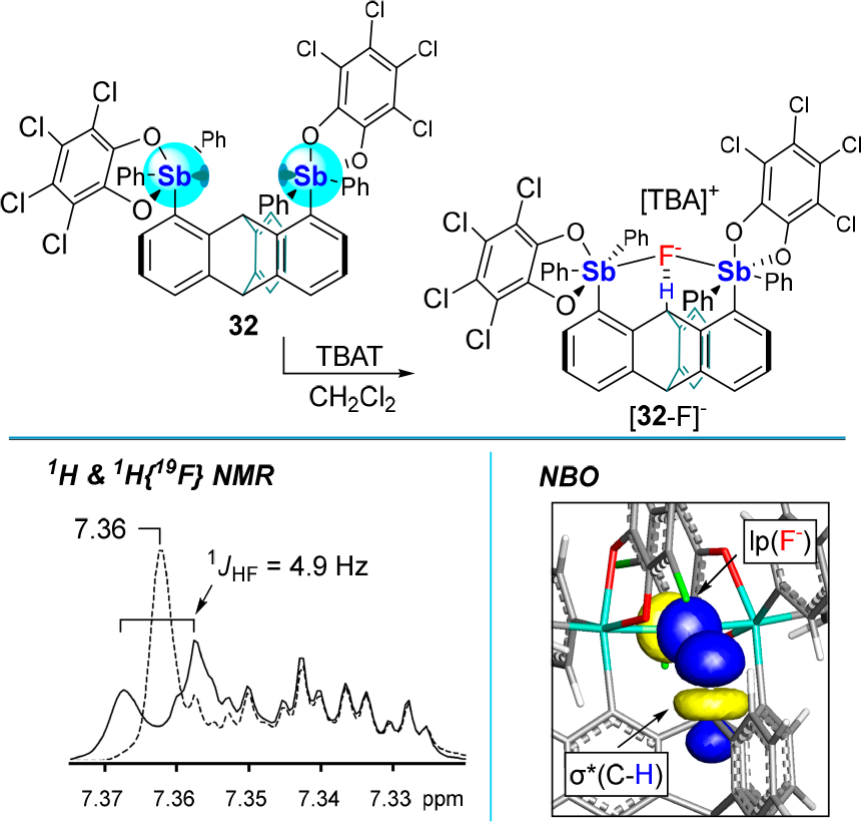
Top: Fluoride complexation by distiborane **32** upon treatment with TBAT in CH_2_Cl_2_. Bottom left: ^1^H and ^1^H{^19^F} NMR showing the coupling of the bridgehead proton to the chelated fluoride in [**32**-F]^−^. Bottom right: Representative NBO of the lp(F^–^) → σ*(C–H) interaction at the triptycene bridgehead.

Despite its promising attributes, fluoride chelation by **30** positions the bound fluoride above the oxygen of the xanthene backbone, leading to a repulsive O···F^–^ interaction that likely dampens the anion affinity of the chelator. To circumvent such a situation, we considered the 1,8-triptycenediyl linker as a backbone whose folded structure would lessen repulsive interactions between the chelated anion and the bridgehead C–H unit. Using standard protocols, we generated the distibine **31** which was easily converted into the distiborane **32** (Scheme 7).^56^ This compound readily complexes a fluoride anion *via* treatment with TBAT in CH_2_Cl_2_ to form [**32**-F][TBA] (Figure 11).^56^ Similar to [**30**-F]^−^, a crystallographic analysis of this complex again shows the two Sb(V) centers clamping down on the bridging fluoride anion that is held by two short Sb–F bonds of 2.158(2) and 2.251(2) Å. Also, the Sb–Sb separation contracts from an average value of 5.20 Å in **32** to 4.3564(5) Å in [**32**-F]^-^.

NBO analysis reveals the presence of an lp(F^–^) → σ*(C_bridgehead_–H) donor–acceptor interaction of 5.9 kJ·mol^–1^ (Figure 11). Though weak, this C–H···F^–^ bond can be clearly observed by ^1^H NMR spectroscopy. The triptycene derivative **32** also has a higher computed FIA value (395 kJ·mol^–1^) than **30** (365 kJ·mol^–1^), suggesting that it is more Lewis acidic . This computational result is corroborated by quantitative fluoride transfer from [**30**-F]^−^ to **32** as confirmed by ^19^F NMR spectroscopy.

**Scheme 8:**
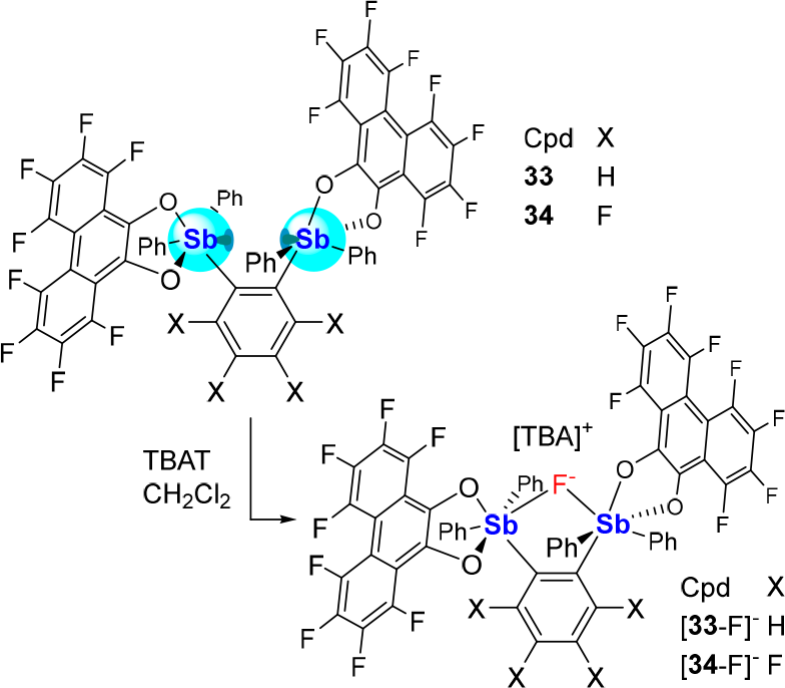
Fluoride Complexation by **33** and **34** upon Treatment with TBAT

**Figure 12:**
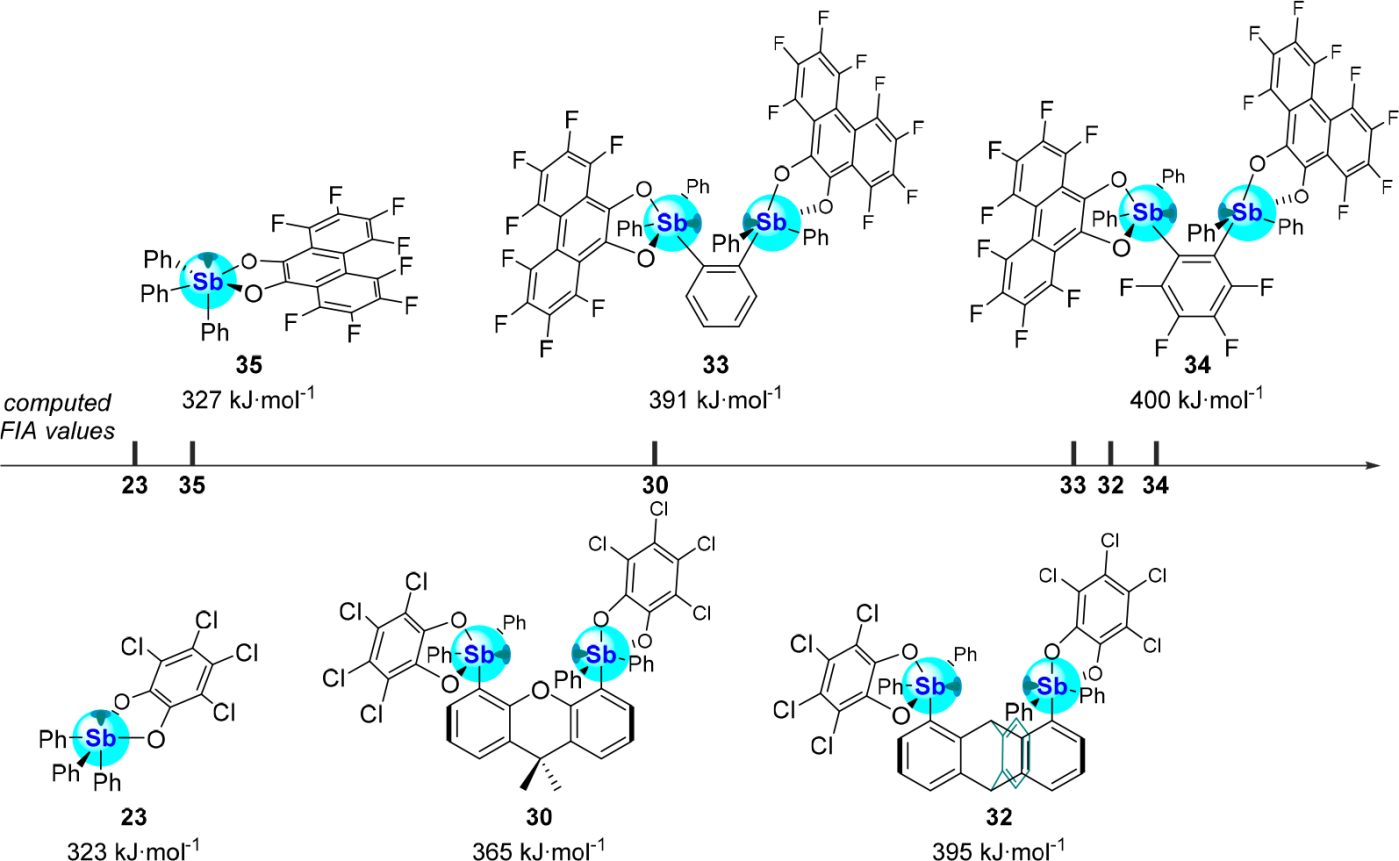
Schematic ranking of the computed gas-phase FIA values of monodentate and bidentate catecholatostiboranes (level of theory: B3LYP/Sb: aug-cc-pVTZ-PP, Cl: 6-311G(d), F: 6-31G(d′), C/O/H: 6-31G).

Our work on these bidentate systems has also explored **33** and **34**, two derivatives based on the *o*-phenylene backbones and featuring the octafluorophenanthrene-9,10-diolate ligand on each antimony, as opposed to the *o*-tetrachlorocatecholate ligand found in **30** and **32** (Scheme 8).^57^ These compounds, which could be accessed using the corresponding distibines following oxidation with 9,10-perfluorophenanthraquinone, differ from one another *via* the nature of the *o*-phenylene backbone, which is perfluorinated in the case of **34**. Each readily complexes fluoride upon addition of TBAT to form the corresponding anionic chelate complexes [**33**-F]^−^ and [**34**-F]^−^, respectively, as [TBA]^+^ salts. In contrast to the roughly linear Sb–F–Sb vectors of [**30**-F]^−^ (165.45(9)°) and [**32**-F]^−^ (174.4(1)°), [**33**-F]^−^ and [**34**-F]^−^ display highly oblique Sb–F–Sb angles of 126.30(12)° and 129.48(6)°, respectively, reflecting the impact of the rigid *o*-phenylene backbone. Gas-phase FIA calculations suggest that **33** (FIA = 391 kJ·mol^–1^) and **34** (FIA = 400 kJ·mol^–1^) are among the most potent fluoride chelators that we have synthesized, with the higher Lewis acidity of **34** being assigned to the perfluorination of the *o*-phenylene backbone (Figure 12). The chelating binding motifs greatly enhance the FIA compared to the monofunctional **35** (FIA = 327 kJ·mol^-1^), further confirming the efficacy of this strategy for anion binding (Figure 12).

### Antimony(V) Compounds – Stibonium Cations

#### Tetraarylstibonium Cations

**Figure 13:**
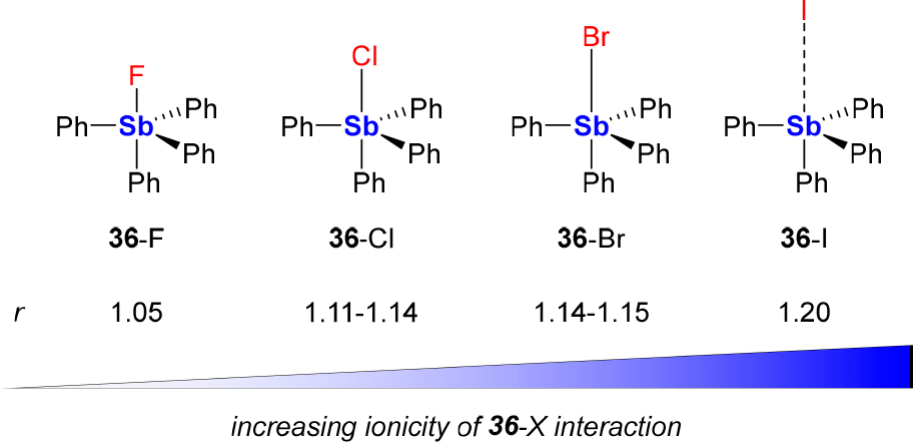
Structures of halide salts of [**36**]^+^ with Sb-X bonds that become increasingly ionic, as depicted by their increasing formal shortness ratio (*r*) values. The *r* values were calculated from published metrical parameters for **36**-F,^58g^**36**-Cl,^58c,64^**36**-Br,^58d,59^ and **36**-I.^61^

Historical precedence has shown that the simplest tetraarylstibonium cation [Ph_4_Sb]^+^ ([**36**]^+^) favors the coordination of small, charge-dense anions^58^ compared to larger anions.^59^ Notably, [**36**]^+^ has long been known to extract aqueous fluoride under biphasic conditions.^13a,60^ Structural analyses of the halide salts of [**36**]^+^ indicate that smaller anions may be privileged for tight complexation by the Lewis acidic antimony center.^61^ Indeed, the solid-state structure of **36**-F displays an Sb–F distance of 2.0530(8) Å,^58g^ which slightly exceeds the sum of the covalent radii of the two elements (R_cov_(Sb) + R_cov_(F) = 1.96 Å).^62^ However, as the halide (X) becomes heavier, the Sb–X bond length increasingly departs from its ideal covalent value, as indicated by the increasing formal shortness ratio (*r* = observed Sb–X bond length/(R_cov_(Sb) + R_cov_(X)), Figure 13).^63^ This increase in *r* reflects the greater ionicity of the Sb–X interaction.

**Scheme 9:**
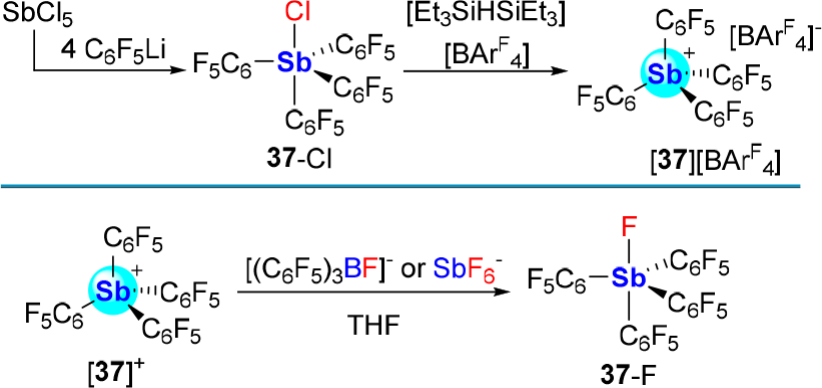
Synthesis of [**37**]^+^ and Fluoride Abstraction Reactions

Our efforts to explore the upper limit of Lewis acidity in tetraarylstibonium cations has led us to consider perfluorinated derivatives.^65^ Toward this end, we prepared (C_6_F_5_)_4_SbCl ([**37**-Cl) by reaction of SbCl_5_ with C_6_F_5_Li.^66^ In line with the increased Lewis acidity imparted to the antimony center by the electron deficient aryl rings, **37**-Cl features an Sb–Cl bond of 2.4509(11) Å, which is much shorter than the values reported for **36**-Cl (2.6860(9) Å and 2.7395(10)).^58c,64^ The high Lewis acidity of [**37**]^+^, which was generated as a [(C_6_F_5_)_4_B]^−^ ([BAr^F^_4_]^−^) salt using **37**-Cl and [Et_3_SiHSiEt_3_][BAr^F^_4_], also manifests in its ability to abstract a fluoride anion from [(C_6_F_5_)_3_BF]^−^ and [SbF_6_]^−^ (Scheme 9), with the last reaction conferring it the distinction of a “Lewis superacid.”^67^ Interestingly, [**37**][SbCl_6_], which can be easily generated from **37**-Cl and SbCl_5_, exists as a stable salt thus highlighting how the antimony center of [**37**]^+^ privileges the smallest halide anion.^68^

**Figure 14:**
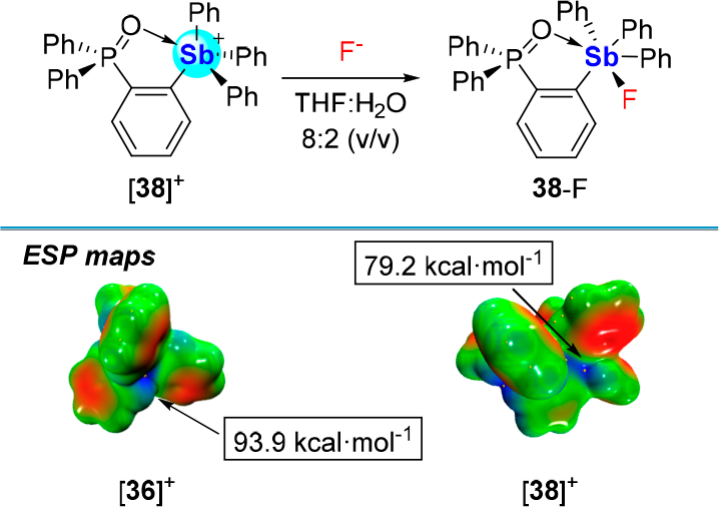
Top: Fluoride binding by [**38**]^+^ in THF:water (8:2 (v/v)). Bottom: ESP maps with arrows indicating the relevant σ holes of [**36**]^+^ and [**38**]^+^ along with the relevant V_S,max_ values (level of theory: B3LYP/Sb: cc-pVTZ with ECP28 MDF, P:6-31+g(d), C/H/O 6-31g).

The anion complexation properties of tetraaryl stibonium centers persist even when near intramolecular Lewis bases.^69^ Such is the case of [**38**]^+^ (Figure 14), which forms a strong intramolecular Lewis interaction with an *ortho* positioned phosphine oxide (Sb–O distance: 2.4315(13) Å).^70^ Even if a comparison of the electrostatic potential (ESP) maps of [**36**]^+^ and [**38**]^+^ indicate partial Lewis acidity quenching due to P=O coordination, [**38**]^+^readily binds fluoride in THF:water (8:2 (v/v)) with a *K*(F^–^) of 10,000 ± 1,000 M^–1^ as determined by ^31^P NMR spectroscopy.

**Scheme 10:**
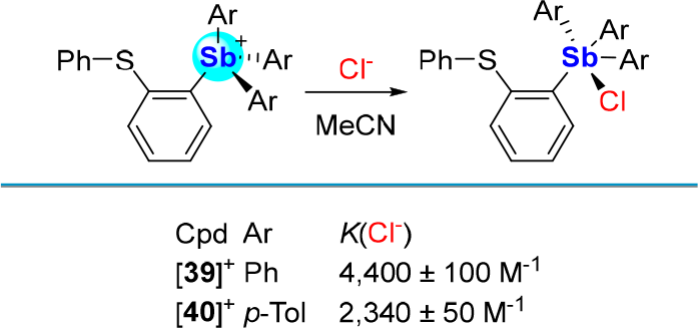
Chloride Binding by [**39**]^+^ and [**40**]^+^ in MeCN

Placement of *o*-phenylthioether moieties on stiboniums [**39**]^+^ and [**40**]^+^ similarly result in somewhat passivating lp(S) → σ*(Sb–C) interactions.^71^ However, because these donor–acceptor interactions are weak, these two cations, which were isolated as tetrafluoroborate salts, retain considerable Lewis acidity. Indeed, chloride titration experiments in MeCN monitored *via* UV–vis spectroscopy finds *K*(Cl^–^) values of 4,400 ± 100 M^–1^ and 2,340 ± 50 M^–1^ for [**39**]^+^ and [**40**]^+^, respectively (Scheme 10). These results suggest that the more electron-releasing *p*-tolyl substituents dampen the Lewis acidity of [**40**]^+^ compared to [**39**]^+^. Such a simple comparison serves as a reminder that the acceptor properties of the antimony center can be finely tuned by variation of its substituents.

**Scheme 11:**
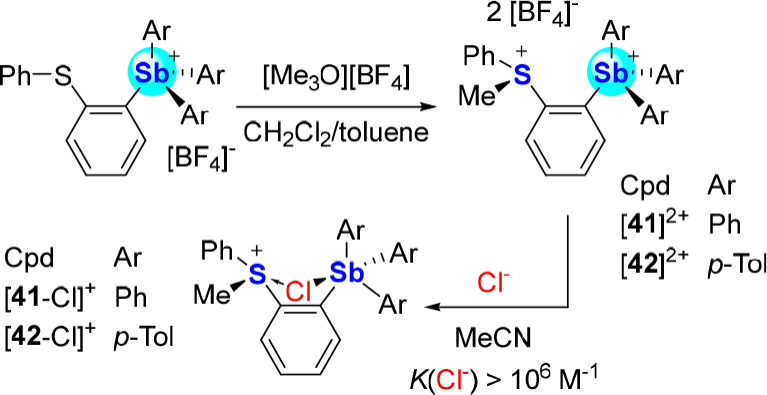
Syntheses of [**41**]^2+^ and [**42**]^2+^ and Chloride Complexation

Positing that the Lewis acidity of the antimony center could be further elevated by alkylation of the thioether functionality, [**39**]^+^ and [**40**]^+^ were treated with [Me_3_O][BF_4_], producing the sulfonium-stibonium dications [**41**]^2+^ and [**42**]^2+^. Both compounds display chloride anion binding constants in excess of 10^6^ M^–1^ in MeCN (Scheme 11), which are several orders of magnitude higher than those of their monocationic precursors [**39**]^+^ and [**40**]^+^.^71^ Computational studies show that the antimony-bound chloride anion of [**41**-Cl]^+^ and [**42**-Cl]^+^ is stabilized by donor–acceptor bonding with the adjacent Lewis acidic sulfonium centers.^72^ Thus, in addition to benefiting from enhanced Coulombic effects, chloride anion binding by [**41**]^2+^ and [**42**]^2+^ may also be enhanced by anion chelation.

#### Triarylmethylstibonium Cations

**Figure 15:**
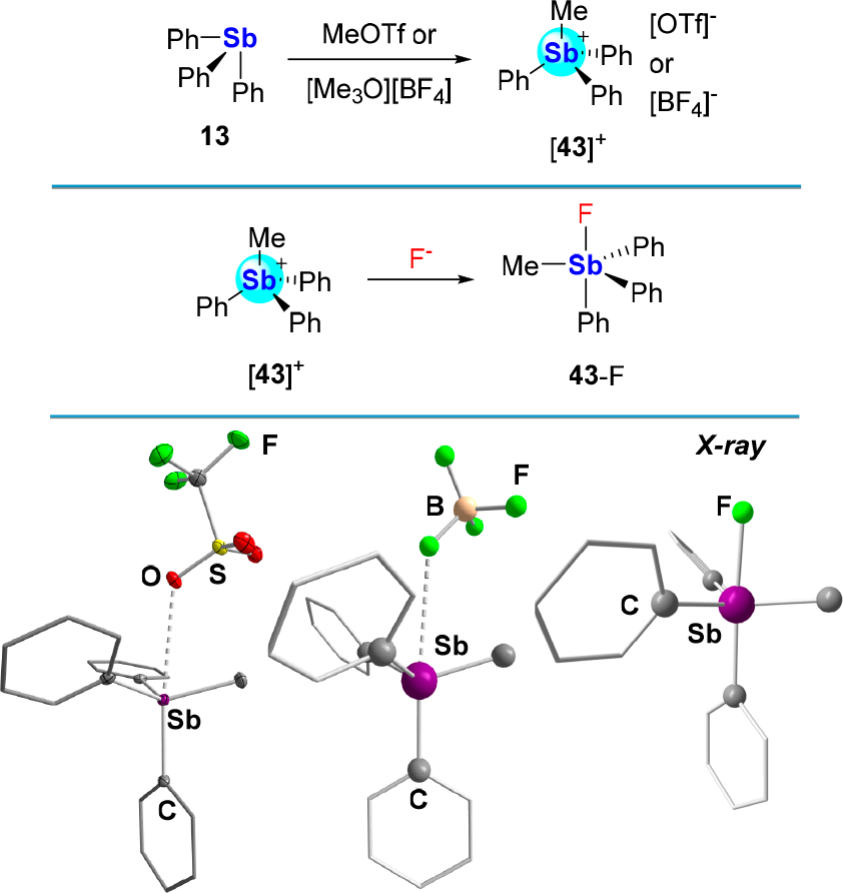
Top: Synthesis of [**43**]^+^ by alkylation of **13** with either MeOTf or [Me_3_O][BF_4_]. Middle: Fluoride complexation by [**43**]^+^. Bottom: Solid-state structures of [**43**][OTf] and [**43**][BF_4_] depicting the short contacts formed between the stibonium centers and the counterions. The solid-state structure of **43**-F is also shown.

Lewis acidity is also evident in the chemistry of the [MePh_3_Sb]^+^ cation ([**43**]^+^), which can be easily accessed by the quaternization of **13** with either MeOTf or [Me_3_O][BF_4_] (Figure 15).^73^ The solid-state structures of the [OTf]^−^ and [BF_4_]^−^ salts of this cation display short contacts with their respective anions (Sb–O distance in [**43**][OTf]: 3.1518(17) Å;^73c^ shortest Sb–F distance in [**43**][BF_4_]: 3.189(10) Å^74^) speaking to the presence of a coordination site *trans* from a phenyl group. This coordination site readily engages a fluoride anion as confirmed by the isolation of **43**-F, which features an Sb–F bond of 2.0687(18) Å^75^ similar to that found for **36**-F.

**Figure 16:**
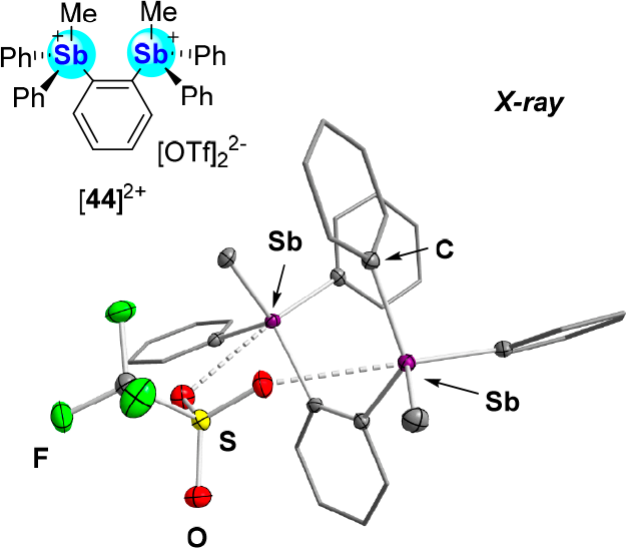
Structure of the dicationic distibonium [**44**][OTf]_2_. The solid-state structure of [**44**]^2+^ depicting the chelation of a [OTf]^−^ counterion across the two stibonium centers is also shown.

Exploiting the simplicity by which triarylmethylstibonium cations can be synthesized, we prepared [**44**]^2+^ as a simple bifunctional analogue and isolated this dication as a bis(triflate) salt ([**44**][OTf]_2_, Figure 16).^73c^ The crystal structure of this salt indicates that one of the triflate counterions bridges the two antimony centers, leading to Sb–O distances of 2.8541(12) and 2.9838(13) Å. These distances are significantly shorter than that found in [**43**][OTf] (3.1518(17) Å), suggesting that the bidentate dication [**44**]^2+^ is a more potent acceptor.

**Figure 17:**
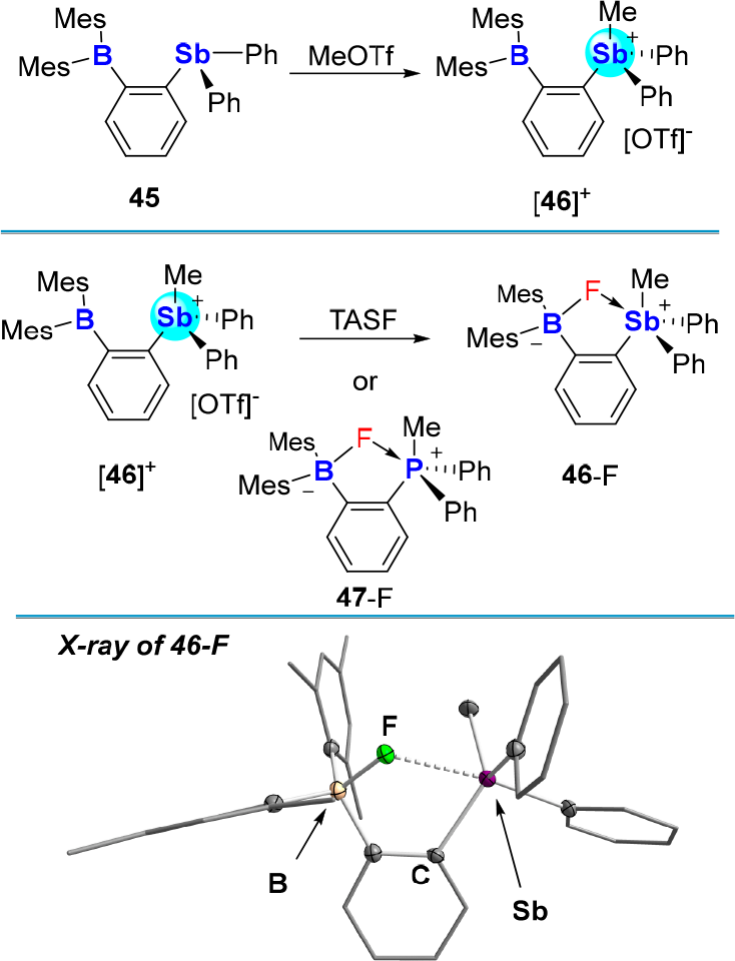
Top: Synthesis of stibonium-borane [**46**]^+^*via* alkylation of stibinoborane **45** with MeOTf. Middle: fluoride chelation by [**46**]^+^*via* TASF or *via* quantitative transfer from **47**-F. Bottom: Solid-state structure of **46**-F showing fluoride chelation between the borane and stibonium Lewis acidic centers.

Anion chelation has also been unambiguously characterized in the case of the heteronuclear stibonium-borane cation [**46**]^+^.^76^ Indeed, this cation, which could be easily synthesized by reaction of the corresponding stibinoborane **45** with MeOTf (Figure 17), forms the corresponding fluoride complex **46**-F upon treatment with [TAS][Me_3_SiF_2_] (TASF). While the ^19^F NMR chemical shift of this complex (−140.1 ppm) is in the range expected for a triarylfluoroborate species,^4c^ its crystal structure shows that the fluoride anion bridges the two Lewis acids, resulting in a B–F bond of 1.521(4) Å and an Sb–F bond of 2.450(2) Å. A competition experiment involving [*o*-(Ph_2_MeP)(Mes_2_B–F)C_6_H_4_] (**47**-F),^77^ the phosphorus analogue of **46-**F, shows quantitative transfer of the fluoride anion to [**46**]^+^ in line with the greater Lewis acidity of the heavier pnictogen. The chelating properties of [**46**]^+^ are also observed in the complexes that this cation forms with azide and cyanide anions.^78^

## Anion Binding Catalysis

### Carbon–Halide Bond Activation

**Figure 18:**
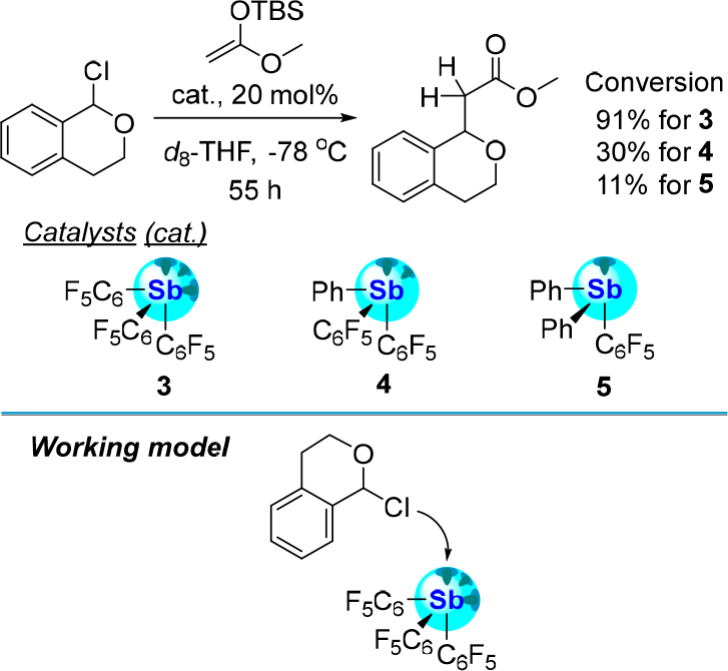
Top: Conversion of 1-chloroisochroman to methyl 2-(isochroman-1-yl)acetate *via* C–Cl activation by stibines **3**–**5**. Bottom: Working model of the catalysis facilitated by **3** depicting the heterolytic dissociation of the C–Br bond at the Lewis acidic stibine center.

Anion binding catalysis has emerged as a popular strategy for the activation of organic substrates whose electrophilic characteristics can be enhanced by the cleavage of a carbon–halide (C–X) bond. This strategy has been successfully implemented using HB donor activators that engage the halide anion and promote the heterolytic dissociation of the C–X bond.^79^ These advances have prompted the exploration of related approaches in which the HB donor activator is replaced by a main group Lewis acid such as a halogen-^80^ or chalcogen-bond donor.^81^ Though more limited, examples also exist in which such reactions have involved PnB donors as activators. Matile and co-workers explored this possibility using stibines **3**, **4**, and **5**, which, as summarized earlier in Figure 3, display an affinity for the chloride anion that scales with the number of pentafluorophenyl groups.^36^ The contrasting anion binding abilities of these compounds also inform their catalytic activities in reactions involving C–Cl bond activations. An example of such a reaction is provided by the activation of 1-chloroisochroman and the generation of an oxocarbenium intermediate that is readily intercepted by a ketene silyl acetal (Figure 18).^36^ The yield of this reaction, after 55 h, increases in lockstep with the number of pentafluorophenyl groups on the stibine center, indicating the favorable influence of the Lewis acidity of the pnictogen bond donor.

**Figure 19:**
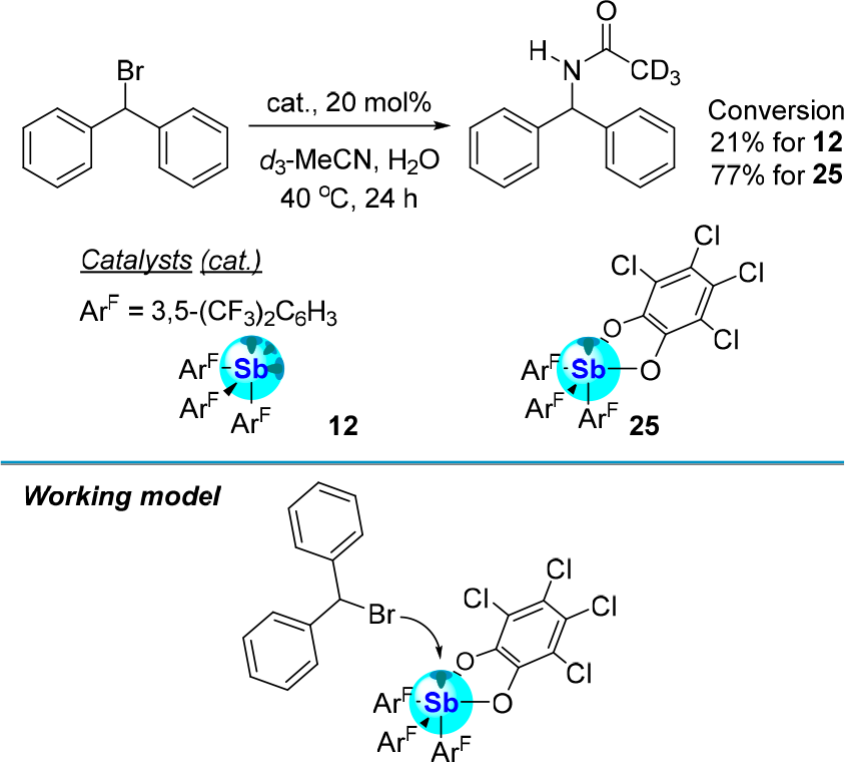
Top: Conversion of diphenylbromomethane to *N*-benzyhydrylacetamide in *d*_3_-MeCN *via* C–Br activation by stibine **12** and catecholatostiborane **25**. Bottom: Working model of the catalysis facilitated by **25** depicting the heterolytic dissociation of the C–Br bond at the Lewis acidic catecholatostiborane center.

Additional studies carried out in our laboratory have explored the role played by the redox state of the antimony center in the Ritter-type reaction of diphenylbromomethane (Figure 19).^19^ This reaction, which necessitates the activation of a C–Br bond, was investigated in CD_3_CN at 40 °C over the course of 24 h using stibine **12** and catecholatostiborane **25** as catalysts. While the reaction proceeded with both catalysts, the conversion obtained with the antimony(V) derivative was markedly higher, as indicated in Figure 19. These results are most simply rationalized by invoking the greater Lewis acidity of the oxidized antimony center of **25**. These results show that the redox state of the antimony atom provides another handle that can be adjusted to augment the catalytic properties of these compounds in reactions that proceed by an anion abstraction step.

### Metal–Chloride Bond Activation

**Figure 20:**
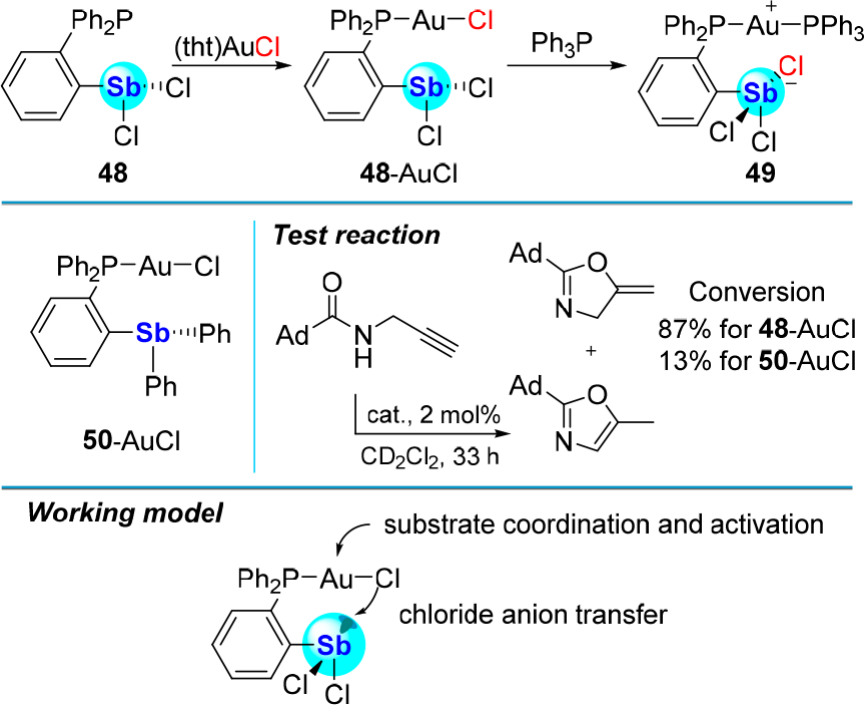
Top: Synthesis of **48**-AuCl *via* auration of **48**, and synthesis of **49** upon addition of Ph_3_P to **48**-AuCl. Middle: Structure of **50**-AuCl, (left), and reaction used to compare the catalytic activities of **48**-AuCl and **50**-AuCl (right). Bottom: Working model developed to rationalize the activity of **48**-AuCl.

The Lewis acid induced heterolysis of metal–halogen (M–X) bonds is a common strategy for the activation of transition metal catalysts.^82^ While this method typically necessitates the use of strong Lewis acids such as fluorinated boranes, recent results have shown that the M–X bond of softer metals can be activated by hydrogen,^83^ halogen,^84^ and chalcogen-bond donor functionalities.^85^ One of our contributions to the development of such approaches has tested whether arylstibine dihalides may be sufficiently chloridophilic to activate M–Cl bonds for application in catalysis. To explore this question, we prepared **48**, a ligand containing a phosphine for metal coordination and a dichlorostibine unit as a chloridophilic PnB donor.^86^ Auration with (tht)AuCl produced compound **48**-AuCl, allowing us to test the possibility of Au–Cl bond activation by the intramolecularly installed dichorostibine functionality. Addition of Ph_3_P produced the trichloroantimonate-containing zwitterion **49**, pointing to the ability of the dichorostibine moiety to indeed participate in Au–Cl bond activation. This conclusion is also supported by the activity of **48**-AuCl as a catalyst for the cyclization of *N*-(prop-2-yn-1-yl)adamantine-1-carboxamide. This reaction, which produced the two isomers shown in Figure 20, proceeded to 87% conversion in 33 h when the reaction was carried out in CD_2_Cl_2_ with 2 mol % catalyst loading. For comparison, we also synthesized complex **50**-AuCl that features a triarylantimony unit as a much less potent PnB donor. This compound proved significantly less active than **48**-AuCl supporting the proposal that the dichlorostibine moiety of **48** activates the gold center, as depicted in the working model in Figure 20.

## Anion Sensing Chemistry

### Neutral Platforms

**Figure 21:**
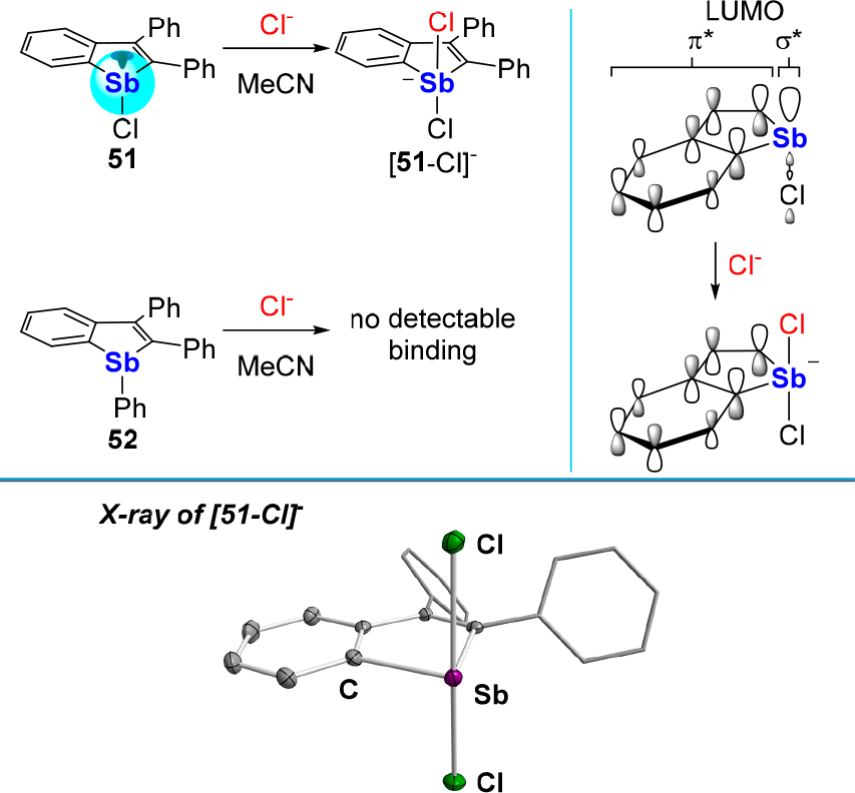
Top left: Chloride anion binding by λ^3^-stibaindoles **51** and **52** in MeCN. Top right: Idealized rendition of the origin of the response *via* the chloride-induced disruption of the σ*/π* conjugation. Bottom: Solid-state structure of [**51**-Cl]^−^.

As noted in the preceding sections, antimony derivatives display elevated anion affinities, leading us to question whether applications in anion sensing could be developed. Working toward this objective, we considered the case of chlorostibines such as Ph_2_SbCl (**2**) that readily complex chloride anions to afford the corresponding [Ph_2_SbCl_2_]^−^ ([**2**-Cl]^−^, Scheme 2). Positing that these simple anion binding events could form the basis of a halide recognition system, we decided to target a chlorostibine, in which the antimony(III) atom is incorporated in a conjugated hydrocarbon π-system. Toward this end, we synthesized the stibaindole **51** as a bright yellow solid.^87^ According to TD-DFT calculations (level of theory: MPW1PW91/Sb: aug-cc-pVTZ-pp, Cl: ECP10MWB, C/H: 6-31g(d)), this compound shows effective conjugation of the π* orbital of the conjugated hydrocarbon backbone and the σ*(Sb–Cl) orbital, which both end up contributing to the LUMO. This compound responds optically to the presence of halide anions including Cl^–^ in MeCN through the loss of its yellow color. This colorimetric response originates from the chloride-induced population of the σ*(Sb–Cl) orbital and the accompanying disruption of the aforementioned σ*−π* conjugation operative in the LUMO (Figure 21). In support of this interpretation, the solid-state structure of [**51**-Cl]^−^ shows that the chloride anion binds to the antimony center *trans* to the chloride ligand along a direction perpendicular to the stibaindole system. A titration experiment carried out in MeCN affords a *K*(Cl^–^) of 95,000 ± 5,000 M^–1^. This elevated binding constant, which is comparable to that of (C_6_F_5_)_3_Sb (**3)**,^36^ speaks to the high Lewis acidity of **51**. This behavior contrasts with that of the phenyl analogue **52**, which shows no evidence of chloride binding under the same conditions. These diverging behaviors illustrate the determining role played by the chloride ligand in its ability to lower the energy of the antimony-centered LUMO while also deepening the corresponding σ-hole.

**Figure 22:**
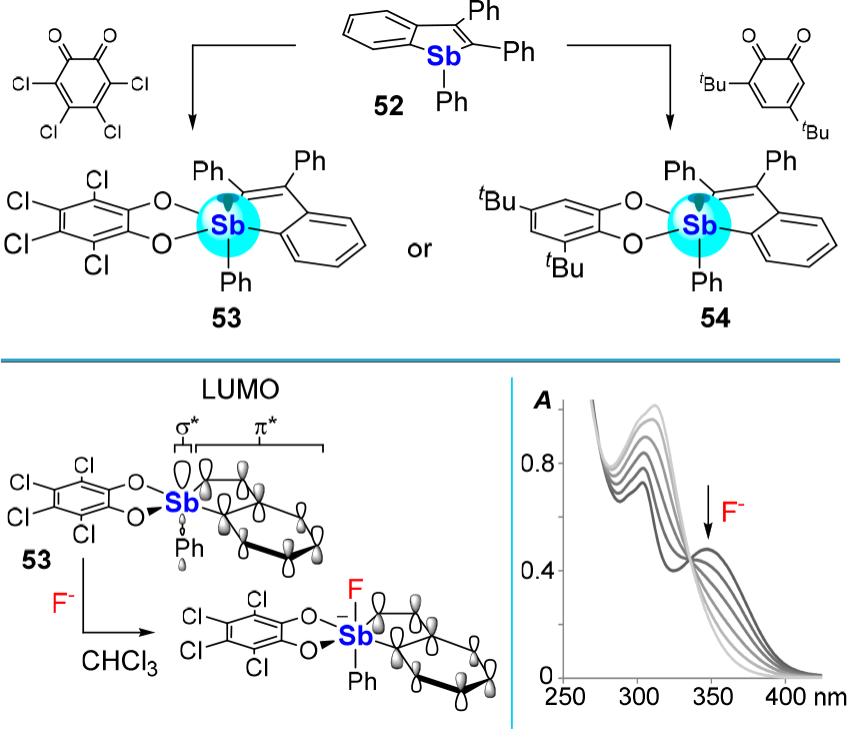
Top: Synthesis of 1λ^5^-stibaindoles **53** and **54** was achieved by oxidation of **52** with *ortho*-quinones. Bottom left: Idealized rendition of the origin of the response *via* fluoride-binding-induced disruption of the σ*/π* conjugation at the LUMO. Bottom right: UV–vis spectra showing the response of **53** to fluoride binding in CHCl_3_.

Contemplating the possibility of anion sensing in aqueous environments and bearing in mind the high hydration energy that typifies anions such as fluoride, we also investigated inherently more Lewis acidic antimony(V) compounds. With this in mind, we generated the bright yellow catecholatostiboranes **53** and **54** by oxidation of **52** with *o*-chloranil and 3,5-di-*tert*-butyl-*o*-benzoquinone, respectively (Figure 22).^88^ The origin of the colors displayed by these stiboranes can be traced back to a stibaindole-based HOMO-1–LUMO transition, resulting in a UV–vis absorption band in the 340–350 nm range, which for both compounds tails into the visible. Interestingly, the stibaindole-based LUMO recruits considerable antimony character as a result of effective conjugation between the σ*(Sb–C_Ph_) orbital and the π* orbital of the hydrocarbon backbone, as shown for **53** in Figure 22. Both **53** and **54** readily bind fluoride in chloroform and, in so doing, lose their bright yellow colors. This turn-off response arises from a disruption of the LUMO that no longer enjoys participation of the σ*(Sb–C_Ph_) orbital, which is now monopolized for binding of the incoming fluoride anion. In THF:water solution (7:3 (v/v)), only **53** engages the fluoride anion, indicating that the more electron poor tetrachlorocatecholate is critical in engendering high Lewis acidity. *Via* DFT calculations (level of theory: B3LYP/Sb: aug-cc-pVTZ-pp, Cl: ECP10MWB, C/H/O: 6-31g(d)), we rationalized these differences as arising from a substantial lowering of the LUMO of **53** (−2.34 eV) compared to that of **54** (−1.84 eV). Finally, in a biphasic CH_2_Cl_2_–H_2_O (pH 4.2, 10 mM citrate, 20 mM tetrapropylammonium bromide) solution, **53** could selectively detect fluoride at levels below 1 ppm.

**Figure 23:**
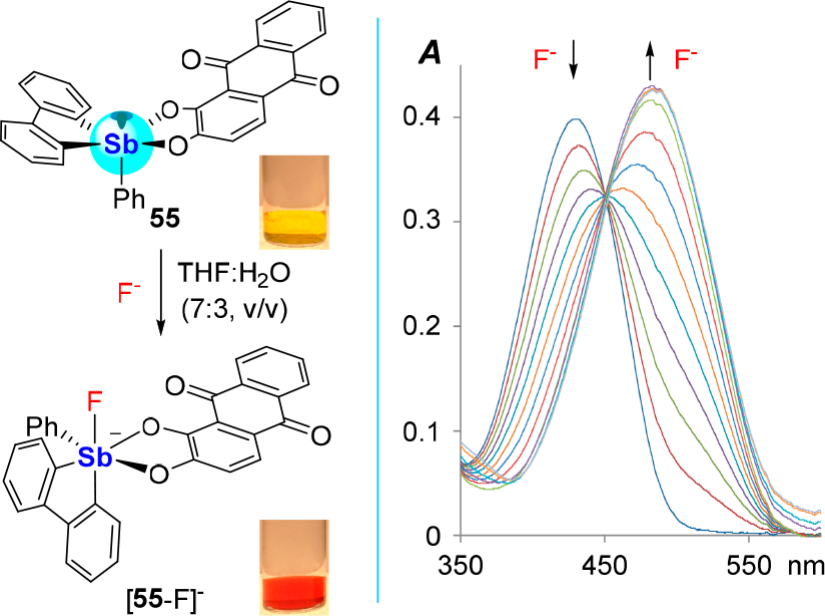
Left: Fluoride binding behavior of by **55** in a THF:water (7:3 (v/v)) solution and colorimetric response to fluoride complexation in CH_2_Cl_2_. Right: UV–vis changes induced by fluoride complexation to **55** in CH_2_Cl_2_.

In another strategy, we considered analogs of the catecholatostiborane **27** (Figure 8), a compound that displays a high affinity for the fluoride anion in aqueous solutions but lacks any distinct photophysical response in the visible part of the spectrum.^53^ Aiming to remediate this situation, we decided to replace the tetrachlorocatecholate ligand by a more performant chromophore such as alizarin,^89^ which could be easily introduced into **55** using standard protocols (Figure 23).^53^ Not only does **55** have a high *K*(F^–^) of 16,100 ± 1,100 M^–1^ in THF:water (7:3 (v/v)), but fluoride binding induces a notable color change from yellow to orange (Figure 23). TD-DFT calculations (level of theory: B3LYP/Sb: aug-cc-pVTZ-PP, F; 6-31g(d′), C/O/H: 6-31g) suggest that this color change is largely driven by an energy increase of the alizarin-based HOMO upon conversion of **55** into the more electron rich fluoroantimonate [**55**-F]^−^. Moreover, an alizarin-based fluorescent turn-on response at 616 nm in dry CH_2_Cl_2_ is also seen between **55** (Φ_F_ = 0.2%) and [**55**-F]^−^ (Φ_F_ = 3.0%), which is attributed to the increased rigidity of the fluoroantimonate (Figure 23). In a biphasic CH_2_Cl_2_–H_2_O (pH 4.68, 10 mM citrate, 20 mM tetrapropylammonium bromide) solution, fluoride binding was selective over Cl^–^, Br^–^, NO_3_^–^, HCO_3_^–^, H_2_PO_4_^–^, and HSO_4_^–^, and was sensitive to fluoride at concentrations less than 1 ppm.

### Cationic Platforms

**Figure 24:**
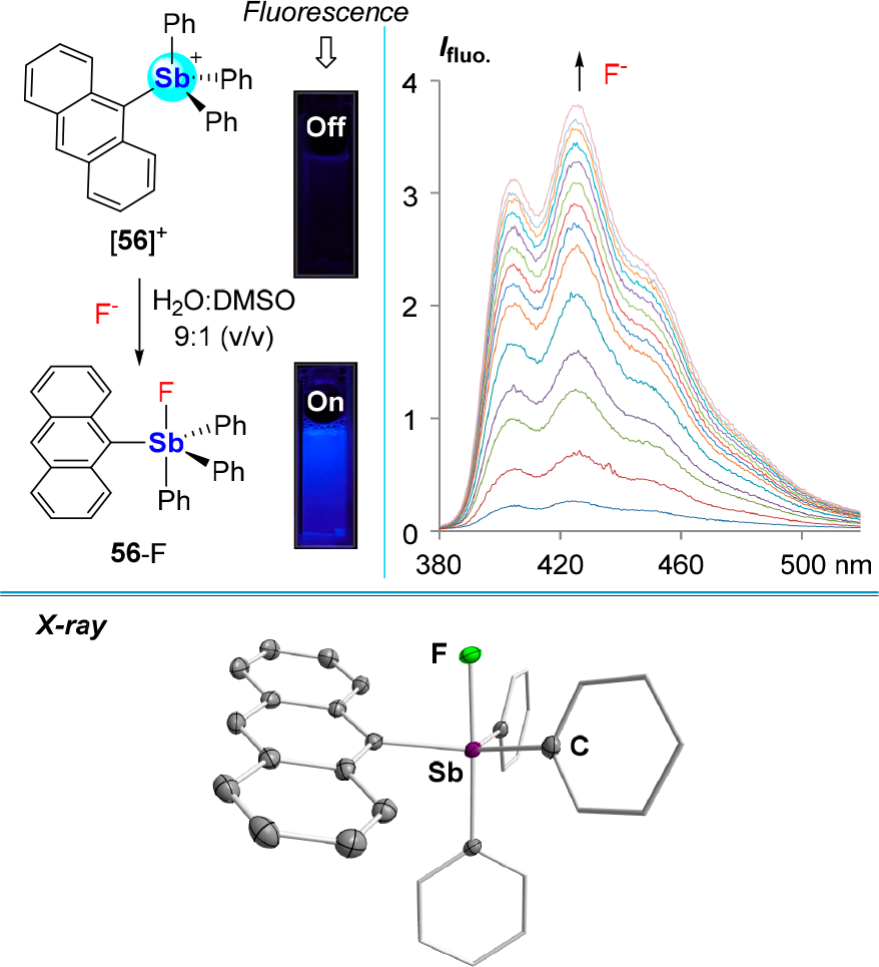
Top left: Fluoride binding by [**56**]^+^ in water:DMSO (9:1 (v/v), pH 4.8, 10 mM pyridine, 10 mM cetyltrimethylammonium bromide), with the fluorescence response to fluoride binding visualized with a hand-held UV lamp. Top right: Fluorescence response to fluoride binding. Bottom: Solid-state structure of **56**-F.

Our earlier work on cationic boron-based anion sensors revealed the determining influence that charge effects can exert over the anion binding at the boron center.^4c,6^ To build on these precedents, and encouraged by the demonstrated ability of [Ph_4_Sb]^+^ ([**36**]^+^) to sequester the fluoride anion under biphasic conditions,^13a,60^ we became eager to investigate the potential of stibonium cations as anion sensors. Since [**36**]^+^ lacks a read-out photophysical response, we replaced one of the phenyl groups by a 9-anthryl group, leading to the isolation of [**56**]^+^ (Figure 24).^90^ This water-compatible stibonium cation is a potent Lewis acid. Because of competitive hydroxide coordination, fluoride binding was studied in a water:DMSO (9:1 (v/v)) solution buffered at pH 4.8. A UV–vis titration experiment carried out under these conditions afforded *K*(F^–^) = 12,000 ± 1,100 M^–1^. To our surprise, fluoride binding by [**56**]^+^ elicited a fluorescence increase from Φ_F_ = 2.2% to Φ_F_ = 14.1% (Figure 24), enabling the use of this stibonium cation as a fluoride anion fluorescence turn-on sensor compatible with subppm concentrations of the anion. Moreover, we found that this response was selective over other anions like Cl^–^, enabling fluoride sensing in tap and bottled water samples.

**Figure 25:**
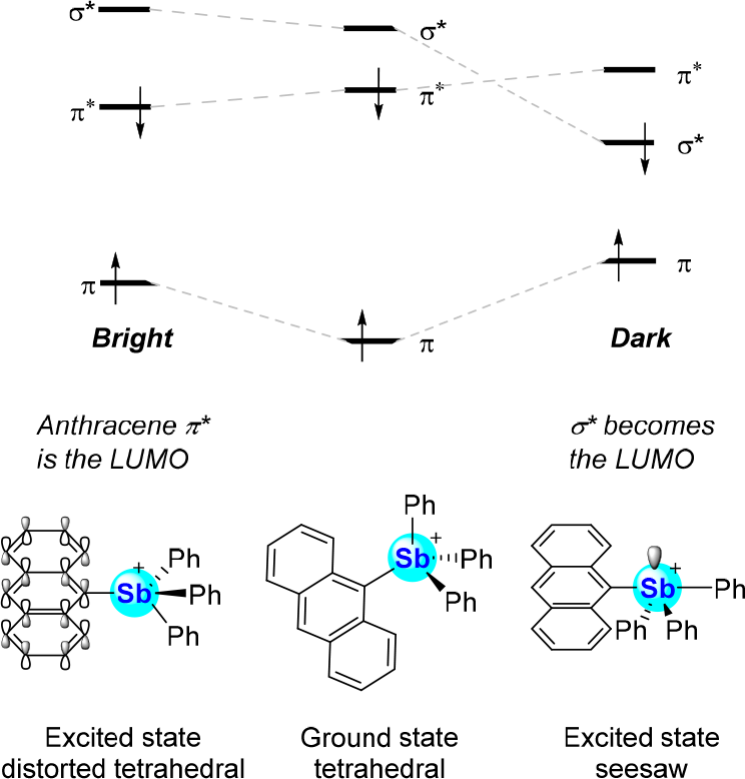
Schematic diagram of the geometry-induced orbital reordering responsible for the emissive properties of [**56**]^+^.

**Figure 26:**
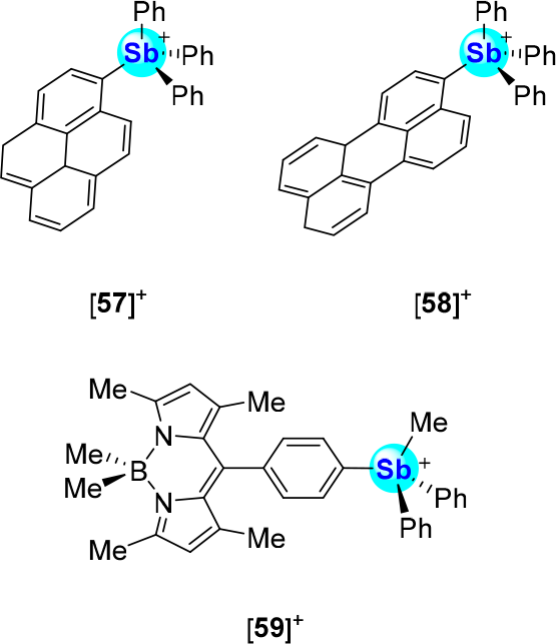
Structures of [**57**]^+^–[**59**]^+^, which undergo fluorescence turn-on responses to fluoride *via* a mechanism similar to [**56**]^+^.

The mechanism of this fluorescence turn-on response was examined by Irle and co-workers *via* excited-state DFT calculations on [**56**]^+^ (level of theory: CAM-B3LYP/Sb: CRENBL, C: 6-311+G(d), H: 6-31G).^91^ Two excited state minima were found in these calculations: an emissive bright state and a nonemissive dark state that lies at a lower energy. In the bright state, the antimony center retains its expected tetrahedral geometry with the HOMO and LUMO corresponding to the π and π* orbitals of the anthryl ligand (Figure 25). The dark state is reached *via* a distortion of the antimony coordination geometry from tetrahedral to seesaw. This distortion, which lowers the energy of the excited state, also changes the nature of the frontier orbitals. The LUMO is particularly affected as it loses its anthryl π* character and relocates on the antimony atom in the form of a σ*(Sb–C) orbital. This distorted excited state is nonemissive, presumably because of the relatively narrow separation between the two SOMOs. Donation of a fluoride lone pair into the σ*(Sb–C) orbital prevents access to such a dark state in **56**-F thus restoring the π–π* emission of the anthracene chromophore. This mechanism also applies to other stibonium cations such as the pyrenyl and perylenyl systems [**57**]^+^ and [**58**]^+^,^92^ and the BODIPY derivative [**59**]^+^ (Figure 26).^93^ All three cations see their fluorescence readily increase upon fluoride binding at the antimony center. In the case of [**57**]^+^, the fluorescence quantum yield increases from Φ_F_ = 0.5% to Φ_F_ = 5.2% upon fluoride binding.^92^ A proportionally similar response is observed for [**58**]^+^, since fluoride binding elicits a fluorescence increase from Φ_F_ = 7.3% to Φ_F_ = 59.2%. Like [**56**]^+^, [**57**]^+^ and [**58**]^+^ are potent fluoride binders with *K*(F^–^) values of 10,000 ± 800 M^–1^ and 10,000 ± 500 M^–1^ for [**57**]^+^ and [**58**]^+^ in a water:DMSO solution (9:1 (v/v), pH 4.8, 10 mM pyridine, 10 mM cetyltrimethylammonium bromide). The BODIPY derivative [**59**]^+^ could only be evaluated in MeCN, a medium in which it displays a *K*(F^–^) greater than 10^7^ M^–1^ and a noticeable fluorescence turn-on response from Φ_F_ = 0.15 to Φ_F_ = 0.30 upon fluoride complexation.^93^ DFT calculations (level of theory: B3LYP/Sb: aug-cc-pVTZ-PP, B/F: 6-31g(d′), C/H/N: 6-31g(d)) suggest that the turn-on response of [**59**]^+^ follows a similar mechanism to that of [**56**]^+^, with anion binding into the σ*(Sb–C_phenylene_) orbital rescuing the π–π*-based emission of the BODIPY fluorophore.^88^

**Figure 27:**
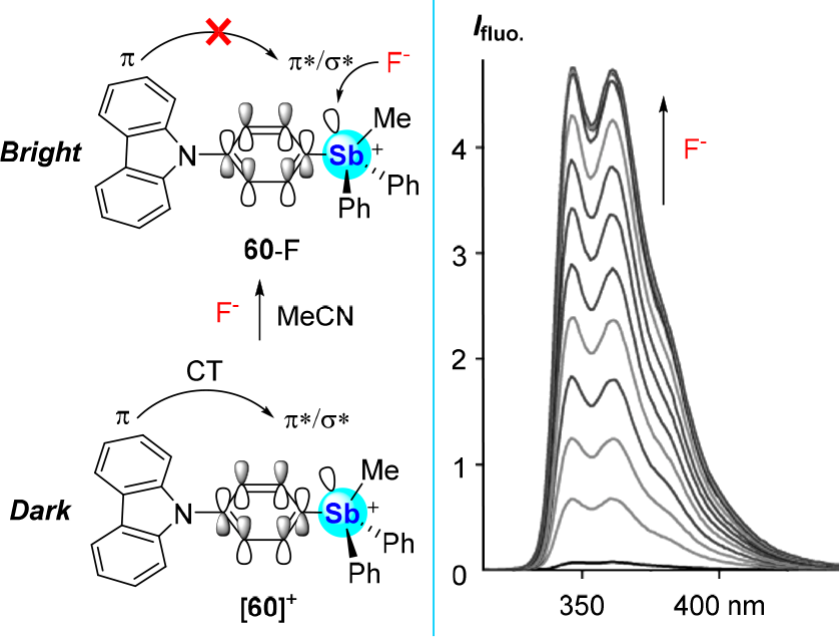
Left: Fluoride binding by [**60**]^+^ in MeCN, with an idealized rendition of the charge-transfer disruption upon fluoride binding. Right: Fluorescence response to fluoride binding in MeCN.

Aiming to replace the electron-poor BODIPY chromophore of [**59**]^+^ by an electron-rich one, we synthesized the carbazole derivative [**60**]^+^ and found it to be very weakly emissive (Φ_F_ = 0.007 in MeCN) (Figure 27).^94^ Addition of TBAF in MeCN affords a *K*(F^–^) > 10^7^ M^–1^, and conversion to **60**-F elicits an order-of-magnitude increase in its quantum yield to Φ_F_ = 0.060. Excited state DFT calculations (level of theory: CAM-B3LYP/Sb: aug-cc-pVTZ-PP with CRENBL ECP, C/H/N/F: 6-31+g(d′)) found that this turn-on mechanism differs from that of [**56**]^+^. While the HOMO and LUMO of **60**-F are confined to the carbazole unit, leading to an emissive π-π* excited state, the LUMO of [**60**]^+^ resides on the antimony-decorated phenylene unit, displaying mixed σ*(Sb–C)/π* character. It follows that, in the case of [**60**]^+^, the classical carbazole-centered π-π* excited state is replaced by an excited state resulting from charge-transfer from the carbazole-based HOMO to the aforementioned σ*(Sb–C)/π* orbital. Experimentally, this excited state is very weakly emissive, possibly because of a narrowed HOMO–LUMO gap.

## Transmembrane Anion Transport by Antimony Compounds

Beyond the realms of catalysis and optical sensing, anion recognition is also essential to a myriad of biological processes. Chief among these processes is the selective transport of anions across hydrophobic membranes, which is typically facilitated by cell-membrane-embedded proteins that form a selective channel to allow for the passage of anions. In so-called channelopathic diseases,^95^ defects in the structure or the expression of these proteins disrupt homeostatic anion transport. Perhaps the best known of these diseases is cystic fibrosis, wherein mutations of the CFTR chloride-bicarbonate anion channel dysregulate its transport activity, leading to a range of harmful consequences.^96^ Investigation into small-molecule treatments for channelopathies has been spent developing “carrier-type” anion transporters that are typically water stable, lipophilic, and adorned with an HB donor binding pocket. Owing to these attributes, such compounds readiliy form anion complexes that can diffuse through lipid bilayers, thus allowing for the transmembrane transport of the anionic cargo.^97^

**Figure 28:**
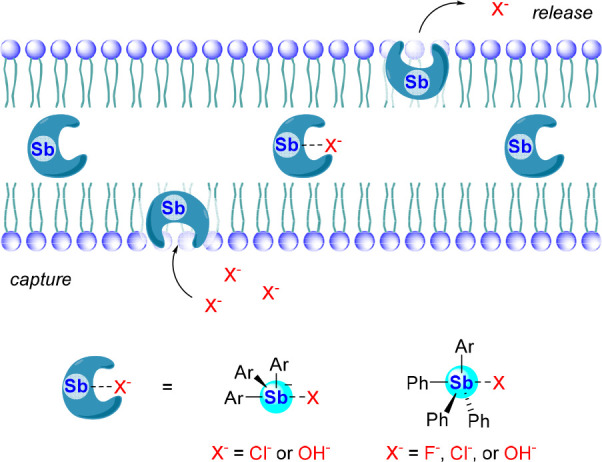
Antimony-based Lewis acids transporting anions across a phospholipid bilayer membrane *via* a carrier-type mechanism.

**Figure 29:**
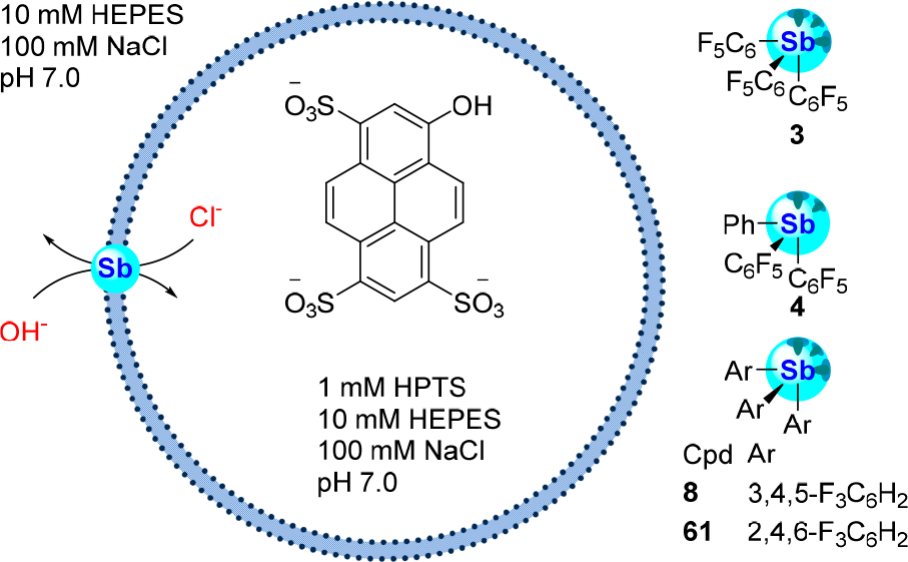
Simplified illustration of the HPTS assay as employed by Matile using EYPC LUVs depicting Cl^–^/OH^–^ antiport upon administration of a base pulse. The structures of stibines **3**, **4**, **8**, and **61** used as transporters are also shown.

The parallels existing between HB-based anion transporters and organoantimony anion binders discussed previously have led our group and that of Matile to test whether electron deficient stibines and stibonium cations could also promote anion transport across phospholipid bilayers *via* a carrier-type mechanism such as that depicted in Figure 28.^98^ As such, the transport activities of Ph(C_6_F_5_)_2_Sb (**4**) were assessed in egg yolk phosphatidylcholine (EYPC) large unilamellar vesicles (LUVs) loaded with the ratiometric pH probe 8-hydropyrene-1,3,6-trisulfonic acid (HPTS) and a buffered solution of NaCl (Figure 29).^99^ Upon administration of a base pulse to the external solution in which the LUVs are suspended, followed by administration of **4**, rapid dissipation of the pH gradient was exhibited *via* Cl^–^/OH^–^ exchange, as indicated by changes in the HPTS fluorescence spectrum.

The EC_50_ value of this transporter, equal to the concentration of the transporter needed to reach 50% chloride transport after 5 min, was estimated to be 1 μM, suggesting that this compound is indeed very active.^99^ This study also established that (C_6_F_5_)_3_Sb (**3**) does not behave ideally, as it also destabilizes the vesicles. A subsequent study also indicated that this molecule is not water stable.^100^ These stability limitations do not seem to affect (3,4,5-C_6_F_3_H_2_)_3_Sb (**8**) and (2,4,6-C_6_F_3_H_2_)_3_Sb (**61**), which were also evaluated using the above-mentioned HPTS assay.^100^ Remarkably, while **61** showed an activity that is moderately improved when compared to that of **4** (EC_50_ = 0.27 ± 0.02 μM for **61** vs 1 μM for **4**), compound **8** displays a much higher activity as indicated by its EC_50_ value of 2.6 ± 0.8 nM, which is 3 orders of magnitude lower than that of **4**. While no explanation for this significant increase between **4** and **8** is provided, it was suggested that **61** is penalized by the *ortho*-fluorine substituents that may hinder the anion binding site or donate to the antimony atom, thereby reducing its Lewis acidity or pnictogen bond donor ability.

**Figure 30:**
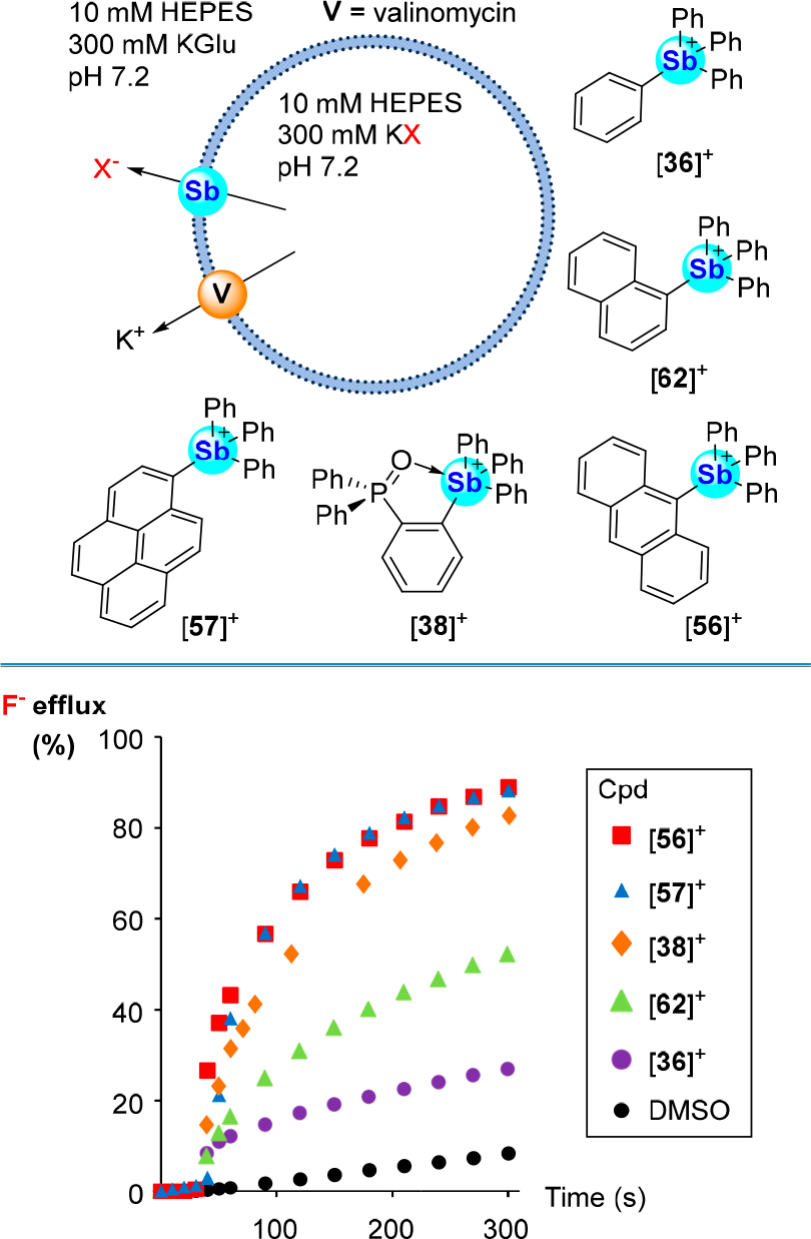
Top: Simplified illustration of the ISE assay as employed by our group using EYPC and POPC LUVs depicting potassium halide salt efflux upon administration of valinomycin and a stibonium cation as transporter. The structures of stibonium cations [**36**]^+^, [**62**]^+^, [**56**]^+^, [**57**]^+^, and [**38**]^+^ used as transporters are also shown. Bottom: Fluoride efflux profiles comparing the transport activities of stibonium cations [**36**]^+^, [**56**]^+^, [**57**]^+^, [**62**]^+^ (in EYPC LUVs), and [**38**]^+^ (in POPC LUVs) added as DMSO solutions, in the presence of valinomycin.

Concomitant with the above efforts, we investigated the anion transport properties of stibonium cations.^101^ These studies, which were encouraged by the known ability of triarylphosphonium cations to permeate through biological membranes,^102^ first focused on tetraarylstibonium cations that were already known to promote anion phase transfer while also serving as water compatible anion sensors.^13a,60,90,92^ We first evaluated [**36**]^+^, [**56**]^+^, [**57**]^+^, and [**62**]^+^ using a fluoride ion selective electrode (ISE) and EYPC LUVs loaded with KF (Figure 30).^101^ We found all four compounds to be potent transporters, either *via* an F^–^/OH^–^ antiport mechanism or *via* KF efflux when administered in the presence of valinomycin as a potassium ion transporter (Figure 30). While all four cations are potent transporters, we observed that the activities of [**56**]^+^ and [**57**]^+^ far exceed those of [**36**]^+^ and [**62**]^+^. These observations led us to propose that the lipophilic character of the stibonium structure assists in partitioning the transporter into the membrane, thus leading to more effective transport. This is supported by computed *n*-octanol/water partition coefficient calculations (log *K*_ow_), which showed that [**56**]^+^ and [**57**]^+^ were at least an order of magnitude more lipophilic (log *K*_ow_ values of 5.85 and 6.26, respectively) than the lower performing [**36**]^+^ and [**62**]^+^ (log *K*_ow_ values of 4.19 and 5.09, respectively). Transport data collected with [**56**]^+^ and [**57**]^+^ in the presence of valinomycin afforded EC_50_ values at 270 s of 0.41 ± 0.05 and 0.57 ± 0.07 mol %, respectively. These EC_50_ values indicate that [**56**]^+^ and [**57**]^+^ rival the transport properties of strapped calix[4]pyrroles investigated *via* fluoride ISE in 1-palmitoyl-2-oleoyl-*sn*-glycero-3-phosphocholine (POPC) vesicles by Gale and co-workers.^103^ We will also note that these values are close to those measured for phosphonium boranes,^104^ which we have also used as fluoride anion transporters. The observation of fluoride transport in the absence of valinomycin provided initial evidence that these stibonium cations may also transport hydroxide anions. We confirmed this possibility using [**36**]^+^, which was deployed in an HPTS assay using EYPC vesicles. This conclusion is reinforced by a recent study employing a lanthanide-based fluoride sensor immobilized inside POPC vesicles to assess fluoride influx.^105^ Using different fluorescence assays, this study concluded that, under the experimental conditions, [**56**]^+^ is indeed a potent hydroxide anion transporter. It follows that [**56**]^+^ may also induce acidification of the vesicle interior and thus formation of HF (p*K*_a_ = 3.17), which can diffuse spontaneously through the membrane. This possibility serves as a reminder that studying the transport of basic anions such as fluoride is inherently complicated.

The fluoride transport properties of stibonium cations do not seem to be affected by the intramolecular coordination of a donor group to the antimony center. Such is the case for the aforementioned [**38**]^+^ whose gas phase FIA is dampened by intramolecular coordination of a phosphine oxide compared to the parent [**36**]^+^ (125.0 kcal·mol^–1^ and 133.0 kcal·mol^–1^, respectively).^70^ Nevertheless, this cation is a very effective transporter, as evidenced by its ability to transport fluoride across the membranes of POPC LUVs (Figure 30). The EC_50_ value of 0.24 ± 0.03 mol % shows that its potency is on par with [**56**]^+^, leading us to propose that the high lipophilicity of [**38**]^+^ (log *K*_ow_ = 7.20) plays a determining role in its performance. These results demonstrate that the transport properties of these stibonium cations survive the introduction of a Lewis basic ligand, providing another dimension along which the composition, properties, and possible conjugability of the transporter could be adjusted.

**Figure 31:**
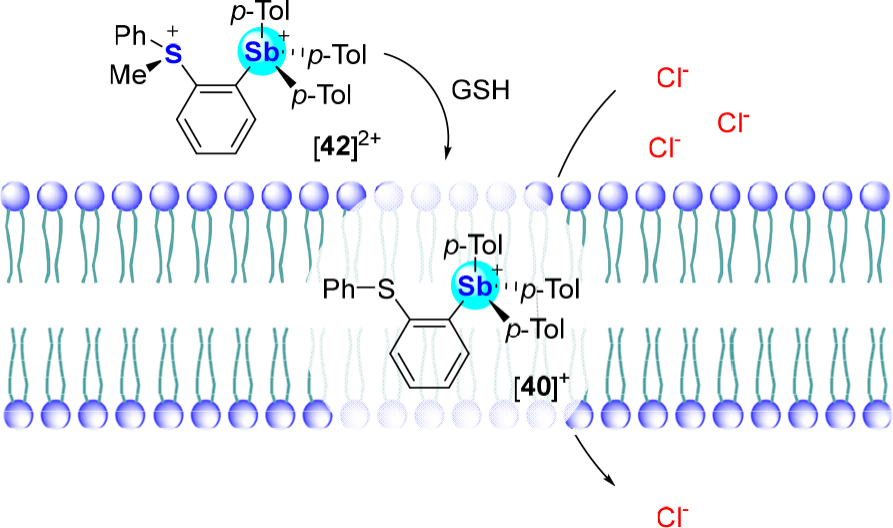
Reductive demethylation of pretransporter [**42**]^2+^ with GSH to yield active chloride transporter [**40**]^+^.

Along similar lines, we observed that the *o*-phenylthioether-stibonium cation [**40**]^+^ is also a very potent anion transporter, as indicated by an assay that employed POPC LUVs loaded with KCl (Figure 31).^71^ Indeed, the EC_50_ of this compound was found to be 0.63 ± 0.03 mol %. The elevated activity of this transporter is correlated to the Lewis acidity of the antimony center as well as the overall lipophilicity of the structure (log *K*_ow_ = 7.68), which promotes recruitment of the transporter to the hydrophobic part of the membrane. As part of this study, we also assayed its corresponding sulfonium-stibonium dication [**42**]^2+^. Even though it is substantially more chloridophilic than [**40**]^+^, [**42**]^2+^ is a significantly less effective transporter. The lower performance of this species is assigned to its decreased lipophilicity (log *K*_ow_ = 1.26), which may limit its ability to partition into the membrane. The contrasting properties of these two compounds led us to hypothesize that the [**40**]^+^/[**42**]^2+^ pair could serve as the basis for a stimulus-responsive transport system. With this idea in mind, we decided to investigate whether [**42**]^2+^ undergoes reduction of the sulfonium center and thus convert into the much more active [**40**]^+^. This possibility was tested using glutathione (GSH), which we first verified cleanly reduces [**42**]^2+^ into [**40**]^+^, in aqueous solutions (D_2_O:*d*_6_-DMSO, 7.9:2.1 (v/v), pH 7.6, 300 mM sodium phosphate) by ^1^H NMR spectroscopy. We also confirmed that the addition of GSH to a solution of KCl-loaded POPC vesicles pretreated with [**42**]^2+^ followed by incubation leads to increased chloride efflux upon administration of valinomycin, as a result of the *in situ* conversion of [**42**]^2+^ into the more active [**40**]^+^. Increasing the concentration of GSH showed a positive correlation with the extent of transport activation, adding further credence to our interpretation (Figure 31).^71^

**Figure 32:**
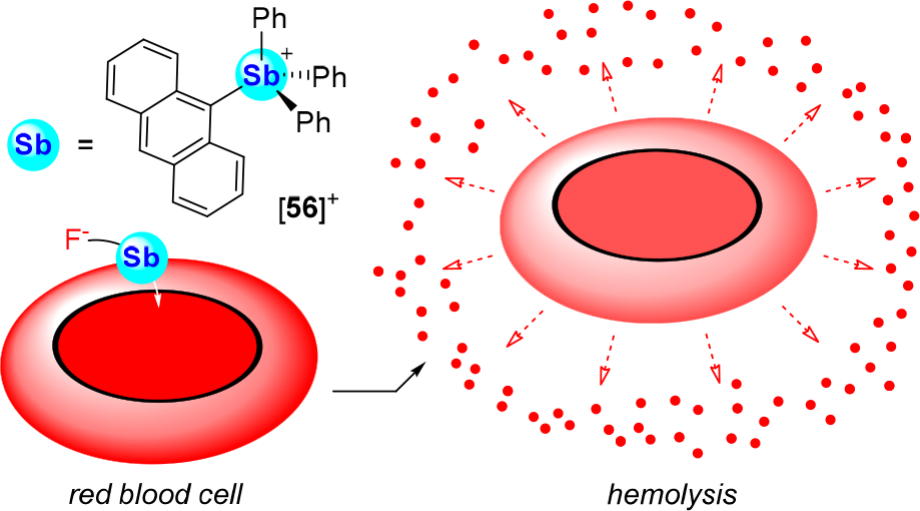
Illustration of the fluoride-induced hemolysis of red blood cells facilitated by fluoride influx by transporter [**56**]^+^.

Finally, with the view of testing stibonium-based anion transporters in biological settings, we decided to test their ability to accelerate the known toxicity of fluoride toward human red blood cells (RBCs), causing oxidative stress and hemolysis when in the presence of fluoride.^106^ Using this toxic side effect as a marker of anion transport, we compared the rate of hemolysis of a sample of RBCs in fluoridated media (100 μM) and [**56**]^+^ (5 μM).^101^ The extent of hemolysis was monitored over time, reaching 48% after 8 h. By comparison, incubation of the RBCs in fluoridated media alone induced only 17% hemolysis after 8 h, while the transporter [**56**]^+^ in the absence of fluoride was found to be nontoxic. The accelerated fluoride-induced toxicity observed in the presence of [**56**]^+^ suggests that the stibonium cation facilitates transport of the fluoride anions inside the RBCs (Figure 32).

## Conclusions and Outlook

The chemistry of organoantimony derivatives has long been limited to structural studies. Over the course of several decades, these studies have revealed the Lewis acidic tendencies that antimony displays especially when bearing electron-withdrawing ligands or when in the pentavalent state. Recent efforts aimed at reclassifying the natural Lewis acidity of antimony derivatives as its ability to form pnictogens bond, or in other words dative bonds with donors have continued to mostly focus on deciphering the structure of the resulting adducts and the nature of the bond connecting the antimony center to the donor. Strikingly, until about 15 years ago, very few efforts had been made to translate the natural Lewis acidity of organoantimony species into a functional context. Our work from the past 12 years, and the contributions of other groups, has begun to change this state of affairs with the demonstration that the Lewis acidity of these main group derivatives can be purposefully exploited for application in anion sensing, anion binding catalysis, and anion transport. The results presented in this Perspective, which focus on our contribution to this area, underscore several important facets, including the ease of synthesis of Lewis acidic organoantimony compounds, their stability toward ambient and aqueous conditions, and the facile tunability of their Lewis acidity, that enable applications in aqueous chemistry where the antimony center is not irreversibly neutralized by water. These group 15 Lewis acids, whose tunability is reminiscent of that of boron-based systems, surpass their group 13 analogs in such conditions .

Thus, the results presented in this Perspective serve as a prelude for antimony’s bright future, specifically in the context of anion binding chemistry. The diverse structural variations that can be implemented to augment anion binding and the simple strategies that can be used to encode photophysical responses provide initial evidence of future possibilities in the field of anion sensing. The same is true in the domain of anion transport chemistry, where stibonium cations have found a new niche as potent carriers of biologically relevant anions across phospholipid membranes. We note though that a deeper understanding of the anion selectivity of these transporters is imperative given that a target anion will constantly compete with hydroxide binding at biological pH values. Attention should also be directed toward expanding the structural breadth of these compounds such that they can toggle their Lewis acidic properties. Clearly the lipophilic character of the transporter influences its anionophoric activity, but the influence of other biological triggers on the transporter structure remains to be determined.

Finally, returning to the fundamental aspects that underpin this chemistry, we will note that while extremely useful the σ hole concept is limited in terms of its ability to predict how anion binding to an antimony center may alter the electronic features of the entire platform. Such a limitation is made particularly obvious by our work on anion sensing, where orbital and electronic structure analyses are necessary to explain the various colorimetric and fluorescence responses that we have observed. Thus, we recommend that the σ hole and σ* orbital models always be paired when considering such chemistry.

## References

[ref1] aPiers W. E.; Chivers T. Pentafluorophenylboranes: from obscurity to applications. Chem. Soc. Rev. 1997, 26, 345–354. 10.1039/cs9972600345.

[ref2] aPiers W. E.; Marwitz A. J. V.; Mercier L. G. Mechanistic Aspects of Bond Activation with Perfluoroarylboranes. Inorg. Chem. 2011, 50, 12252–12262. 10.1021/ic2006474. 21612200

[ref3] Lee M. H.; Agou T.; Kobayashi J.; Kawashima T.; Gabbaï F. P. Fluoride ion complexation by a cationic borane in aqueous solution. Chem. Commun. 2007, 1133–1135. 10.1039/b616814k. 17347716

[ref4] aAgou T.; Sekine M.; Kobayashi J.; Kawashima T. Detection of Biologically Important Anions in Aqueous Media by Dicationic Azaborines Bearing Ammonio or Phosphonio Groups. Chem.—Eur. J. 2009, 15, 5056–5062. 10.1002/chem.200802159. 19326374

[ref5] Chiu C.-W.; Kim Y.; Gabbaï F. P. Lewis Acidity Enhancement of Triarylboranes via Peripheral Decoration with Cationic Groups. J. Am. Chem. Soc. 2009, 131, 60–61. 10.1021/ja808572t. 19093849

[ref6] Zhao H.; Leamer L. A.; Gabbaï F. P. Anion capture and sensing with cationic boranes: on the synergy of Coulombic effects and onium ion-centred Lewis acidity. Dalton Trans 2013, 42, 8164–8178. 10.1039/c3dt50491c. 23599021

[ref7] Hudnall T. W.; Gabbaï F. P. Ammonium Boranes for the Selective Complexation of Cyanide or Fluoride Ions in Water. J. Am. Chem. Soc. 2007, 129, 11978–11986. 10.1021/ja073793z. 17845043

[ref8] Lippert A. R.; Van de Bittner G. C.; Chang C. J. Boronate oxidation as a bioorthogonal reaction approach for studying the chemistry of hydrogen peroxide in living systems. Acc. Chem. Res. 2011, 44, 793–804. 10.1021/ar200126t. 21834525 PMC3178007

[ref9] Baaz M.; Gutmann V.; Kunze O. Die Chloridionenaffinitäten von Akzeptorchloriden in Acetonitril, 1. Mitt.: Lösungszustand und Bildungsgleichgewichte der Triphenylchlormethankomplexe. Monatsh. Chem. 1962, 93, 1142–1161. 10.1007/BF00905917.

[ref10] aOlah G. A.; Kuhn S. J.; Tolgyesi W. S.; Baker E. B. Stable Carbonium Ions. II. Oxocarbonium (Acylium) Tetrafluoroborates, Hexafluorophosphates, Hexafluoroantimonates and Hexafluoroarsenates. Structure and Chemical Reactivity of Acyl Fluoride: Lewis Acid Fluoride Complexes. J. Am. Chem. Soc. 1962, 84, 2733–2740. 10.1021/ja00873a019.

[ref11] aCalves J. Y.; Gillespie R. J. The methyl fluoride-antimony pentafluoride and arsenic pentafluoride systems: the formation of methyl fluoride.antimony pentafluoride and methyl fluoride.arsenic pentafluoride and the methylation of thionyl fluoride and sulfuryl chloride fluoride. J. Am. Chem. Soc. 1977, 99, 1788–1792. 10.1021/ja00448a018.

[ref12] Olah G. A.; Prakash G. K. S.; Molnár Á.; Sommer J. Superacid chemistry, 2nd ed.; Wiley: Hoboken, NJ, 2009.

[ref13] aMoffett K. D.; Simmler J. R.; Potratz H. A. Solubilities of Tetraphenylstibonium Salts of Inorganic Anions. Procedure For Solvent Extraction of Fluoride Ion From Aqueous Medium. Anal. Chem. 1956, 28, 1356 10.1021/ac60116a047.

[ref14] aLin T.-P.; Wade C. R.; Pérez L. M.; Gabbaï F. P. A Mercury→Antimony Interaction. Angew. Chem., Int. Ed. 2010, 49, 6357–6360. 10.1002/anie.201002995. 20661984

[ref15] aWade C. R.; Ke I.-S.; Gabbaï F. P. Sensing of Aqueous Fluoride Anions by Cationic Stibine–Palladium Complexes. Angew. Chem. Int. Ed 2012, 51, 478–481. 10.1002/anie.201106242. 22113959

[ref16] Gutmann V.; Hubacek H.; Steininger A. Color indicators in proton-free solutions. III. Crystal Violet in phosphorus oxychloride. Monatsh. Chem. 1964, 95, 678–686. 10.1007/BF00908782.

[ref17] van Zeist W.-J.; Bickelhaupt F. M. The activation strain model of chemical reactivity. Org. Biomol. Chem. 2010, 8, 3118–3127. 10.1039/b926828f. 20490400

[ref18] de Azevedo Santos L.; Hamlin T. A.; Ramalho T. C.; Bickelhaupt F. M. The pnictogen bond: a quantitative molecular orbital picture. Phys. Chem. Chem. Phys. 2021, 23, 13842–13852. 10.1039/D1CP01571K. 34155488 PMC8297534

[ref19] Yang M.; Tofan D.; Chen C.-H.; Jack K. M.; Gabbaï F. P. Digging the Sigma-Hole of Organoantimony Lewis Acids by Oxidation. Angew. Chem., Int. Ed. 2018, 57, 13868–13872. 10.1002/anie.201808551. 30151881

[ref20] aErdmann P.; Leitner J.; Schwarz J.; Greb L. An Extensive Set of Accurate Fluoride Ion Affinities for p-Block Element Lewis Acids and Basic Design Principles for Strong Fluoride Ion Acceptors. ChemPhysChem 2020, 21, 987–994. 10.1002/cphc.202000244. 32212357 PMC7317340

[ref21] Maltz L. T.; Gabbaï F. P. Analyzing Fluoride Binding by Group 15 Lewis Acids: Pnictogen Bonding in the Pentavalent State. Inorg. Chem. 2023, 62, 13566–13572. 10.1021/acs.inorgchem.3c019. 37551938 PMC10862541

[ref22] Varadwaj A.; Varadwaj P. R.; Marques H. M.; Yamashita K. Definition of the Pnictogen Bond: A Perspective. Inorganics 2022, 10, 149 10.3390/inorganics10100149.

[ref23] Cavallo G.; Metrangolo P.; Milani R.; Pilati T.; Priimagi A.; Resnati G.; Terraneo G. The Halogen Bond. Chem. Rev. 2016, 116, 2478–2601. 10.1021/acs.chemrev.5b00484. 26812185 PMC4768247

[ref24] Wang W.; Ji B.; Zhang Y. Chalcogen bond: a sister noncovalent bond to halogen bond. J. Phys. Chem. A 2009, 113, 8132–5. 10.1021/jp904128b. 19537765

[ref25] Bauzá A.; Mooibroek T. J.; Frontera A. The Bright Future of Unconventional σ/π-Hole Interactions. ChemPhysChem 2015, 16, 2496–2517. 10.1002/cphc.201500314. 26118675

[ref26] Alcock N. W., Secondary Bonding to Nonmetallic Elements. In Advances in Inorganic Chemistry and Radiochemistry; Emeléus H. J., Sharpe A. G., Eds.; Academic Press: 1972; Vol. 15, pp 1–58.

[ref27] Norman N. C. Coordination Chemistry of Antimony and Bismuth: Lewis Acidity, σ*-Orbitals and Coordination Geometry. Phosphorus, Sulfur, and Silicon and the Related Elements 1994, 87, 167–176. 10.1080/10426509408037451.

[ref28] Scheiner S. A new noncovalent force: Comparison of P···N interaction with hydrogen and halogen bonds. J. Chem. Phys. 2011, 134, 094315 10.1063/1.3562209. 21384977

[ref29] aHimmel D.; Krossing I.; Schnepf A. Dative Bonds in Main-Group Compounds: A Case for Fewer Arrows!. Angew. Chem., Int. Ed. 2014, 53, 370–374. 10.1002/anie.201300461. 24243854

[ref30] Mundt O.; Becker G.; Stadelmann H.; Thurn H. Element—Element-Bindungen. VII. Intermolekulare Wechselwirkungen bei Dihalogen(phenyl)stibanen. Z. Anorg. Allg. Chem. 1992, 617, 59–71. 10.1002/zaac.19926170110.

[ref31] aHall M.; Sowerby D. B. Phenylchloroantimon(III)ates; their preparations, and the crystal structures of Me_4_N[PhSbCl_3_], [Hpy]_2_[PhSbCl_4_], and Me_4_N[Ph_2_SbCl_2_]. J. Organomet. Chem. 1988, 347, 59–70. 10.1016/0022-328X(88)80269-9.

[ref32] aCarmalt C. J.; Cowley A. H.; Culp R. D.; Jones R. A.; Kamepalli S.; Norman N. C. Synthesis and Structures of Intramolecularly Base-Coordinated Group 15 Aryl Halides. Inorg. Chem. 1997, 36, 2770–2776. 10.1021/ic9701165. 11669910

[ref33] Becker G.; Mundt O.; Sachs M.; Breunig H. J.; Lork E.; Probst J.; Silvestru A. Element-element bonds. X. Studies of chloro(diphenyl)stibane, tribenzylstibane and tribenzyldibromostiborane - Molecular structures and isotypism. Z. Anorg. Allg. Chem. 2001, 627, 699–714. 10.1002/1521-3749(200104)627:4<699::AID-ZAAC699>3.0.CO;2-U.

[ref34] Calderazzo F.; Marchetti F.; Ungari F.; Wieber M. Reactivity of Molecules Containing Element-Element Bonds. 3. Reduction of Halo-Organometallics of Antimony(III) and Bismuth(III) - Crystal and Molecular-Structure of CoCp_2_[SbPh_2_Cl_2_]. Gazz. Chim. Ital. 1991, 121, 93–100.

[ref35] aLevason W.; McAuliffe C. A. Coordination chemistry of organostibines. Acc. Chem. Res. 1978, 11, 363–8. 10.1021/ar50129a007.

[ref36] Benz S.; Poblador-Bahamonde A. I.; Low-Ders N.; Matile S. Catalysis with pnictogen, chalcogen, and halogen bonds. Angew. Chem. Int. Ed 2018, 57, 5408–5412. 10.1002/anie.201801452. PMC5947745 29558562

[ref37] Kuhn H.; Docker A.; Beer P. D. Anion Recognition with Antimony(III) and Bismuth(III) Triaryl-Based Pnictogen Bonding Receptors. Chem.—Eur. J. 2022, 28, e202201838 10.1002/chem.202201838. 35968660 PMC10092038

[ref38] aQiu J.; Unruh D. K.; Cozzolino A. F. Design, Synthesis, and Structural Characterization of a Bisantimony(III) Compound for Anion Binding and the Density Functional Theory Evaluation of Halide Binding through Antimony Secondary Bonding Interactions. J. Phys. Chem. A 2016, 120, 9257–9269. 10.1021/acs.jpca.6b08170. 27768303

[ref39] Qiu J.; Bateman C. N.; Lu S.; George G. C. III; Li X.; Gorden J. D.; Vasylevskyi S.; Cozzolino A. F. Solution Studies of a Water-Stable, Trivalent Antimony Pnictogen Bonding Anion Receptor with High Binding Affinities for CN^–^, OCN^–^, and OAc^–^. Inorg. Chem. 2023, 62, 12582–12589. 10.1021/acs.inorgchem.3c01887. 37499143

[ref40] Zaitseva E. G.; Medvedev S. V.; Aslanov L. A. Crystal and Molecular-Structures of Cesium Phenylpentachloroantimonate Cs[PhSbCl_5_], Potassium Phenylpentabromoantimonate K[PhSbBr_5_], and Cesium Hexachloroantimonate Cs[SbCl_6_]. J. Struct. Chem. 1990, 31, 92–97. 10.1007/BF00752019.

[ref41] aNunn M.; Begley M. J.; Sowerby D. B.; Haiduc I. Complexes of organoantimony(III) and (V) halides with nitrogen donors. Polyhedron 1996, 15, 3167–3174. 10.1016/0277-5387(96)00055-1.

[ref42] Karimi M.; Gabbaï F. P. Hydrogen Bond-Assisted Fluoride Binding by a Stiborane. Z. Anorg. Allg. Chem. 2022, 648, e202200098 10.1002/zaac.202200098.

[ref43] Kasemann R.; Naumann D. Tieftemperatur-flüssigphasefluorierung von pentafluorphenyl-element-Verbindungen. Darstellungen und eigenschaften von (C_6_F_5_)_3_AsF_2_, (C_6_F_5_)_3_SbF_2_, (C_6_F_5_)_2_SeF_2_, (C_6_F_5_)_2_SeO, C_6_F_5_TeF_3_ und Cs[(C_6_F_5_)_3_EF_3_] (E = As, Sb) [1]. J. Fluorine Chem. 1988, 41, 321–334. 10.1016/S0022-1139(00)81033-7.

[ref44] Kojima S.; Doi Y.; Okuda M.; Akiba K. Y. First Stereochemical Characterization of Configurationally Stable Diastereomers of Hypervalent Stiboranes (10-Sb-5) and Acceleration of Intramolecular Permutation by Donor Solvents. Organometallics 1995, 14, 1928–1937. 10.1021/om00004a053.

[ref45] Holmes R. R.; Day R. O.; Chandrasekhar V.; Holmes J. M. Pentacoordinated Molecules. 67. Formation and Structure of Cyclic 5-Coordinated Antimony Derivatives - the First Square-Pyramidal Geometry for a Bicyclic Stiborane. Inorg. Chem. 1987, 26, 157–163. 10.1021/ic00248a031.

[ref46] Hirai M.; Gabbaï F. P. Squeezing Fluoride out of Water with a Neutral Bidentate Antimony(V) Lewis Acid. Angew. Chem. Int. Ed 2015, 54, 1205–1209. 10.1002/anie.201410085. 25424599

[ref47] Kruger G. J.; Pistorius C. W. F. T.; Heyns A. M. Potassium hexafluoroantimonate (I). Acta Crystallogr., Sect. B: Struct. Sci. 1976, 32, 2916–2918. 10.1107/S0567740876009230.

[ref48] Gini A.; Paraja M.; Galmés B.; Besnard C.; Poblador-Bahamonde A. I.; Sakai N.; Frontera A.; Matile S. Pnictogen-bonding catalysis: brevetoxin-type polyether cyclizations. Chem. Sci. 2020, 11, 7086–7091. 10.1039/D0SC02551H. 33250977 PMC7690316

[ref49] Chishiro A.; Akioka I.; Sumida A.; Oka K.; Tohnai N.; Yumura T.; Imoto H.; Naka K. Tetrachlorocatecholates of triarylarsines as a novel class of Lewis acids. Dalton Trans 2022, 51, 13716–13724. 10.1039/D2DT02145E. 36004500

[ref50] aLiberman-Martin A. L.; Bergman R. G.; Tilley T. D. Lewis Acidity of Bis(perfluorocatecholato)silane: Aldehyde Hydrosilylation Catalyzed by a Neutral Silicon Compound. J. Am. Chem. Soc. 2015, 137, 5328–5331. 10.1021/jacs.5b02807. 25879515 PMC4428610

[ref51] Roth D.; Wadepohl H.; Greb L. Bis(perchlorocatecholato)germane: Hard and Soft Lewis Superacid with Unlimited Water Stability. Angew. Chem., Int. Ed. 2020, 59, 20930–20934. 10.1002/anie.202009736. PMC7693072 32776679

[ref52] Roth D.; Stirn J.; Stephan D. W.; Greb L. Lewis Superacidic Catecholato Phosphonium Ions: Phosphorus–Ligand Cooperative C–H Bond Activation. J. Am. Chem. Soc. 2021, 143, 15845–15851. 10.1021/jacs.1c07905. 34521202

[ref53] Hirai M.; Gabbaï F. P. Lewis acidic stiborafluorenes for the fluorescence turn-on sensing of fluoride in drinking water at ppm concentrations. Chem. Sci. 2014, 5, 1886–1893. 10.1039/C4SC00343H.

[ref54] Smith J. E.; Gabbaï F. P. Are Ar_3_SbCl_2_ Species Lewis Acidic? Exploration of the Concept and Pnictogen Bond Catalysis Using a Geometrically Constrained Example. Organometallics 2023, 42, 240–245. 10.1021/acs.organomet.2c00565. 38333362 PMC10848295

[ref55] aKatz H. E. Recent advances in multidentate anion complexation. Inclusion Compd 1991, 4, 391–405.

[ref56] Chen C.-H.; Gabbaï F. P. Fluoride Anion Complexation by a Triptycene-Based Distiborane: Taking Advantage of a Weak but Observable C–H···F Interaction. Angew. Chem., Int. Ed. 2017, 56, 1799–1804. 10.1002/anie.201611009. 28067453

[ref57] You D.; Zhou B.; Hirai M.; Gabbaï F. P. Distiboranes based on *ortho*-phenylene backbones as bidentate Lewis acids for fluoride anion chelation. Org. Biomol. Chem. 2021, 19, 4949–4957. 10.1039/D1OB00536G. 33988214

[ref58] aBeauchamp A. L.; Bennett M. J.; Cotton F. A. Molecular structure of tetraphenylantimony hydroxide. J. Am. Chem. Soc. 1969, 91, 297–301. 10.1021/ja01030a015.

[ref59] Ferguson G.; Glidewell C.; Lloyd D.; Metcalfe S. Effect of the counter ion on the structures of tetraphenylantimony(V)-stibonium compounds: crystal and molecular structures of tetraphenylantimony(V) bromide, perchlorate, and tetraphenylborate. J. Chem. Soc., Perkin Trans. 2 1988, 731–735. 10.1039/P29880000731.

[ref60] aBowen L. H.; Rood R. T. Solvent extraction of ^18^F as tetraphenylstibonium fluoride. J. Inorg. Nucl. Chem. 1966, 28, 1985–1990. 10.1016/0022-1902(66)80290-7.

[ref61] Baker L.-J.; Rickard C. E. F.; Taylor M. J. Structural investigations of the organoantimony(V) halides Ph_4_SbX and Ph_3_SbX_2_ (X = Cl, Br or I) in the solid state and in solution. Dalton Trans 1995, 2895–2899. 10.1039/dt9950002895.

[ref62] Cordero B.; Gómez V.; Platero-Prats A. E.; Revés M.; Echeverría J.; Cremades E.; Barragán F.; Alvarez S. Covalent radii revisited. Dalton Trans 2008, 2832–2838. 10.1039/b801115j. 18478144

[ref63] Cotton F. A. Discovering and understanding multiple metal-to-metal bonds. Acc. Chem. Res. 1978, 11, 225–232. 10.1021/ar50126a001.

[ref64] Sharutin V. V.; Sharutina O. K.; Pakusina A. P.; Platonova T. P.; Zadachina O. P.; Gerasimenko A. V. Phenylation of antimony(V) organic compounds with pentaphenylantimony. The structure of tetraphenylantimony chloride. Russ. J. Coord. Chem. 2003, 29, 89–92. 10.1023/A:1022377815791.

[ref65] aCoughlin O.; Krämer T.; Benjamin S. L. Cationic Triarylchlorostibonium Lewis Acids. Organometallics 2023, 42, 339–346. 10.1021/acs.organomet.2c00426. 36937787 PMC10015551

[ref66] Pan B.; Gabbaï F. P. [Sb(C_6_F_5_)_4_][B(C_6_F_5_)_4_]: An Air Stable, Lewis Acidic Stibonium Salt That Activates Strong Element-Fluorine Bonds. J. Am. Chem. Soc. 2014, 136, 9564–9567. 10.1021/ja505214m. 24946107

[ref67] Greb L. Lewis Superacids: Classifications, Candidates, and Applications. Chem.—Eur. J. 2018, 24, 17881–17896. 10.1002/chem.201802698. 29943864

[ref68] Yang M.; Pati N.; Bélanger-Chabot G.; Hirai M.; Gabbaï F. P. Influence of the catalyst structure in the cycloaddition of isocyanates to oxiranes promoted by tetraarylstibonium cations. Dalton Trans 2018, 47, 11843–11850. 10.1039/C8DT00702K. 29697133

[ref69] Arias Ugarte R.; Devarajan D.; Mushinski R. M.; Hudnall T. W. Antimony(V) cations for the selective catalytic transformation of aldehydes into symmetric ethers, α,β-unsaturated aldehydes, and 1,3,5-trioxanes. Dalton Trans 2016, 45, 11150–11161. 10.1039/C6DT02121B. 27326797

[ref70] Gonzalez V. M.; Park G.; Yang M.; Gabbaï F. P. Fluoride anion complexation and transport using a stibonium cation stabilized by an intramolecular P=O → Sb pnictogen bond. Dalton Trans 2021, 50, 17897–17900. 10.1039/D1DT03370K. 34816847

[ref71] Park G.; Gabbaï F. P. Redox-controlled chalcogen and pnictogen bonding: the case of a sulfonium/stibonium dication as a preanionophore for chloride anion transport. Chem. Sci. 2020, 11, 10107–10112. 10.1039/D0SC04417B. 34094272 PMC8162396

[ref72] Kim Y.; Kim M.; Gabbaï F. P. Synthesis and Anion Affinity of a Bidendate Sulfonium Fluorosilane Lewis Acid. Org. Lett. 2010, 12, 600–602. 10.1021/ol902641v. 20052988

[ref73] aHenry M. C.; Wittig G. The Organometallic Alkylidene Reaction. J. Am. Chem. Soc. 1960, 82, 563–564. 10.1021/ja01488a017.

[ref74] Low J. N.; Ferguson G.; Wardell J. L. Methyltriphenylstibonium tetrafluoroborate. Acta Crystallogr., Sect. C: Cryst. Struct. Commun. 2000, 56, e317 10.1107/S0108270100008945.

[ref75] Bordner J.; Andrews B. C.; Long G. G. Fluoro(methyl)triphenylantimony, C_19_H_18_FSb. Cryst. Struct. Commun. 1976, 5, 801–804.

[ref76] Wade C. R.; Gabbaï F. P. Fluoride Anion Chelation by a Bidentate Stibonium–Borane Lewis Acid. Organometallics 2011, 30, 4479–4481. 10.1021/om200499y.

[ref77] Hudnall T. W.; Kim Y.-M.; Bebbington M. W. P.; Bourissou D.; Gabbaï F. P. Fluoride ion chelation by a bidentate phosphonium/borane Lewis acid. J. Am. Chem. Soc. 2008, 130, 10890–10891. 10.1021/ja804492y. 18652460

[ref78] Wade C. R.; Gabbaï F. P. Cyanide and Azide Anion Complexation by a Bidentate Stibonium-Borane Lewis Acid. Z. Naturforsch., B: J. Chem. Sci. 2014, 69, 1199–1205. 10.5560/znb.2014-4168.

[ref79] aBrak K.; Jacobsen E. N. Asymmetric Ion-Pairing Catalysis. Angew. Chem., Int. Ed. 2013, 52, 534–561. 10.1002/anie.201205449. PMC4284951 23192886

[ref80] Bulfield D.; Huber S. M. Halogen Bonding in Organic Synthesis and Organocatalysis. Chem.—Eur. J. 2016, 22, 14434–14450. 10.1002/chem.201601844. 27465662

[ref81] Vogel L.; Wonner P.; Huber S. M. Chalcogen bonding: an overview. Angew. Chem., Int. Ed. 2019, 58, 1880–1891. 10.1002/anie.201809432. 30225899

[ref82] aChen E. Y.-X.; Marks T. J. Cocatalysts for metal-catalyzed olefin polymerization: activators, activation processes, and structure-activity relationships. Chem. Rev. 2000, 100, 1391–1434. 10.1021/cr980462j. 11749269

[ref83] aSen S.; Gabbaï F. P. An ambiphilic phosphine/H-bond donor ligand and its application to the gold mediated cyclization of propargylamides. Chem. Commun. 2017, 53, 13356–13358. 10.1039/C7CC06065C. 29199294

[ref84] aWolf J.; Huber F.; Erochok N.; Heinen F.; Guérin V.; Legault C. Y.; Kirsch S. F.; Huber S. M. Activation of a Metal-Halogen Bond by Halogen Bonding. Angew. Chem. Int. Ed 2020, 59, 16496–16500. 10.1002/anie.202005214. PMC7540446 32472957

[ref85] Zhou B.; Gabbaï F. P. Anion Chelation via Double Chalcogen Bonding: The Case of a Bis-telluronium Dication and Its Application in Electrophilic Catalysis via Metal–Chloride Bond Activation. J. Am. Chem. Soc. 2021, 143, 8625–8630. 10.1021/jacs.1c04482. 34085823

[ref86] Jones J. S.; Gabbaï F. P. Activation of an Au-Cl Bond by a Pendent Sb^III^ Lewis Acid: Impact on Structure and Catalytic Activity. Chem.—Eur. J. 2017, 23, 1136–1144. 10.1002/chem.201604521. 27813226

[ref87] Christianson A. M.; Gabbaï F. P. A Lewis Acidic, π-Conjugated Stibaindole with a Colorimetric Response to Anion Binding at Sb(III). Organometallics 2017, 36, 3013–3015. 10.1021/acs.organomet.7b00419.

[ref88] Christianson A. M.; Rivard E.; Gabbaï F. P. 1λ^5^-Stibaindoles as Lewis acidic, π-conjugated, fluoride anion responsive platforms. Organometallics 2017, 36, 2670–2676. 10.1021/acs.organomet.7b00289.

[ref89] Kubo Y.; Kobayashi A.; Ishida T.; Misawa Y.; James T. D. Detection of anions using a fluorescent alizarin-phenylboronic acid ensemble. Chem. Commun. 2005, 2846–2848. 10.1039/b503588k. 15928778

[ref90] Ke I.-S.; Myahkostupov M.; Castellano F. N.; Gabbaï F. P. Stibonium Ions for the Fluorescence Turn-On Sensing of F^–^ in Drinking Water at Parts per Million Concentrations. J. Am. Chem. Soc. 2012, 134, 15309–15311. 10.1021/ja308194w. 22954306

[ref91] Usui K.; Ando M.; Yokogawa D.; Irle S. Understanding the On–Off Switching Mechanism in Cationic Tetravalent Group-V-Based Fluoride Molecular Sensors Using Orbital Analysis. J. Phys. Chem. A 2015, 119, 12693–12698. 10.1021/acs.jpca.5b09709. 26647787

[ref92] Hirai M.; Myahkostupov M.; Castellano F. N.; Gabbaï F. P. 1-Pyrenyl- and 3-Perylenyl-antimony(V) Derivatives for the Fluorescence Turn-On Sensing of Fluoride Ions in Water at Sub-ppm Concentrations. Organometallics 2016, 35, 1854–1860. 10.1021/acs.organomet.6b00233.

[ref93] Christianson A. M.; Gabbaï F. P. Anion sensing with a Lewis acidic BODIPY-antimony(V) derivative. Chem. Commun. 2017, 53, 2471–2474. 10.1039/C6CC09205E. 28180216

[ref94] Kumar A.; Yang M.; Kim M.; Gabbaï F. P.; Lee M. H. OFF–ON Fluorescence Sensing of Fluoride by Donor–Antimony(V) Lewis Acids. Organometallics 2017, 36, 4901–4907. 10.1021/acs.organomet.7b00759.

[ref95] Kim J.-B. Channelopathies. Korean J. Pediatr. 2014, 57, 1–18. 10.3345/kjp.2014.57.1.1. 24578711 PMC3935107

[ref96] Ratjen F.; Bell S. C.; Rowe S. M.; Goss C. H.; Quittner A. L.; Bush A. Cystic fibrosis. Nat. Rev. Dis. Primers 2015, 1, 15010 10.1038/nrdp.2015.10. 27189798 PMC7041544

[ref97] aDavis A. P.; Sheppard D. N.; Smith B. D. Development of synthetic membrane transporters for anions. Chem. Soc. Rev. 2007, 36, 348–357. 10.1039/B512651G. 17264935 PMC2854546

[ref98] Docker A.; Langton M. J. Transmembrane anion transport mediated by sigma-hole interactions. Trends Chem. 2023, 10.1016/j.trechm.2023.06.001.

[ref99] Lee L. M.; Tsemperouli M.; Poblador-Bahamonde A. I.; Benz S.; Sakai N.; Sugihara K.; Matile S. Anion transport with pnictogen bonds in direct comparison with chalcogen and halogen bonds. J. Am. Chem. Soc. 2019, 141, 810–814. 10.1021/jacs.8b12554. 30618243

[ref100] Humeniuk H. V.; Gini A.; Hao X.; Coelho F.; Sakai N.; Matile S. Pnictogen-Bonding Catalysis and Transport Combined: Polyether Transporters Made In Situ. JACS Au 2021, 1, 1588–1593. 10.1021/jacsau.1c00345. 34723261 PMC8549043

[ref101] Park G.; Brock D. J.; Pellois J.-P.; Gabbaï F. P. Heavy Pnictogenium Cations as Transmembrane Anion Transporters in Vesicles and Erythrocytes. Chem. 2019, 5, 2215–2227. 10.1016/j.chempr.2019.06.013. 31482145 PMC6719792

[ref102] Smith R. A. J.; Hartley R. C.; Cocheme H. M.; Murphy M. P. Mitochondrial pharmacology. Trends Pharmacol. Sci. 2012, 33, 341–352. 10.1016/j.tips.2012.03.010. 22521106

[ref103] Clarke H. J.; Howe E. N.; Wu X.; Sommer F.; Yano M.; Light M. E.; Kubik S.; Gale P. A. Transmembrane Fluoride Transport: Direct Measurement and Selectivity Studies. J. Am. Chem. Soc. 2016, 138, 16515–16522. 10.1021/jacs.6b10694. 27998094

[ref104] Park G.; Gabbaï F. P. Phosphonium Boranes for the Selective Transport of Fluoride Anions across Artificial Phospholipid Membranes. Angew. Chem., Int. Ed. 2020, 59, 5298–5302. 10.1002/anie.201914958. 31945248

[ref105] Cataldo A.; Chvojka M.; Park G.; Šindelář V.; Gabbaï F. P.; Butler S. J.; Valkenier H. Transmembrane transport of fluoride studied by time-resolved emission spectroscopy. Chem. Commun. 2023, 59, 4185–4188. 10.1039/D3CC00897E. PMC10072081 36938842

[ref106] Agalakova N. I.; Gusev G. P. Fluoride-induced death of rat erythrocytes*in**vitro*. Toxicol. in Vitro 2011, 25, 1609–1618. 10.1016/j.tiv.2011.06.006. 21704696

